# Ultrafiltration separation of Am(VI)-polyoxometalate from lanthanides

**DOI:** 10.1038/s41586-023-05840-z

**Published:** 2023-04-19

**Authors:** Hailong Zhang, Ao Li, Kai Li, Zhipeng Wang, Xiaocheng Xu, Yaxing Wang, Matthew V. Sheridan, Han-Shi Hu, Chao Xu, Evgeny V. Alekseev, Zhenyi Zhang, Pu Yan, Kecheng Cao, Zhifang Chai, Thomas E. Albrecht-Schönzart, Shuao Wang

**Affiliations:** 1 State Key Laboratory of Radiation Medicine and Protection, School for Radiological and Interdisciplinary Sciences (RAD-X) and Collaborative Innovation Center of Radiation Medicine of Jiangsu Higher Education Institutions, Soochow University, Suzhou, China; 2 Institute of Nuclear and New Energy Technology, Tsinghua University, Beijing, China; 3 Department of Chemistry and Laboratory of Organic Optoelectronics & Molecular Engineering of the Ministry of Education, Tsinghua University, Beijing, China; 4 IEK-9, Forschungszentrum Jülich, Jülich, Germany; 5 Bruker (Beijing) Scientific Technology Co., Ltd, Shanghai, China; 6 Shanghai Key Laboratory of High-resolution Electron Microscopy, ShanghaiTech University, Shanghai, China; 7 Department of Chemistry and Nuclear Science & Engineering Center, Colorado School of Mines, Golden, CO USA

**Keywords:** Nuclear chemistry, Nuclear waste

## Abstract

Partitioning of americium from lanthanides (Ln) present in used nuclear fuel plays a key role in the sustainable development of nuclear energy^1–3^. This task is extremely challenging because thermodynamically stable Am(III) and Ln(III) ions have nearly identical ionic radii and coordination chemistry. Oxidization of Am(III) to Am(VI) produces AmO_2_^2+^ ions distinct with Ln(III) ions, which has the potential to facilitate separations in principle. However, the rapid reduction of Am(VI) back to Am(III) by radiolysis products and organic reagents required for the traditional separation protocols including solvent and solid extractions hampers practical redox-based separations. Herein, we report a nanoscale polyoxometalate (POM) cluster with a vacancy site compatible with the selective coordination of hexavalent actinides (^238^U, ^237^Np, ^242^Pu and ^243^Am) over trivalent lanthanides in nitric acid media. To our knowledge, this cluster is the most stable Am(VI) species in aqueous media observed so far. Ultrafiltration-based separation of nanoscale Am(VI)-POM clusters from hydrated lanthanide ions by commercially available, fine-pored membranes enables the development of a once-through americium/lanthanide separation strategy that is highly efficient and rapid, does not involve any organic components and requires minimal energy input.

## Abstract (web summary)

A new strategy to separate radioactive americium from lanthanides based on complexation with polyoxometalates and ultrafiltration technique is highly efficient and rapid, does not involve any organic components and requires minimal energy input.

## Main

Americium is a neutron-capture by-product of nuclear power generation and a major contributor to the long-term radiotoxicity of high-level waste. The efficient recovery of americium followed by transmutation into short-lived or stable nuclides using fast reactors would significantly reduce the environmental impact of nuclear energy. However, the coexistence of lanthanides (Ln) with high neutron capture cross-sections (for example, ^157^Gd) severely limits transmutation efficiency. Overcoming this impediment requires the development of efficient separations between americium and lanthanides and has remained a long-standing challenge in the nuclear industry for decades. This difficulty originates primarily from their similar chemical behaviour because both americium and lanthanides exist in solution as thermodynamically stable trivalent cations that possess nearly identical ionic radii and coordination chemistry. Traditional separations exploit the subtle bonding differences between Am(III) and Ln(III) ions whereby extractants containing nitrogen or sulfur donors enable preferential partitioning of Am(III) over Ln(III)^4,5^. This separation strategy, however, is still hampered by limited discrimination between Am(III) and Ln(III), and, more notably, by the generation of large amounts of secondary radioactive liquid waste.

One proposed method for mitigating this separation challenge is the oxidation of Am(III) to the higher oxidation states of Am(V) and Am(VI)^6^. These cations possess coordination chemistry that parallels the linear dioxo early actinyl ions, such as UO_2_^2+^ and NpO_2_^+^, with anisotropic coordination contrasting sharply with relatively isotropic Ln(III) ions^7^. This, in principle, leads to better discrimination between americium and lanthanides and a subsequent increase in separation efficiency. Although various techniques have been explored following redox-based protocol, including solvent extraction^8–11^, precipitation^12^ and ion-exchange chromatography^13^, an unsolved issue is unavoidable reduction of high-valent Am back to Am(III) during the separation process. Am(VI) cations are strong oxidizing agents with reduction potentials of 1.6 V and 1.68 V for AmO_2_^2+^/AmO_2_^+^ and AmO_2_^2+^/Am^3+^ couples, respectively (versus saturated calomel electrode (SCE))^6^. Therefore, Am(III) species can be produced in a few seconds once Am(VI) ions contact organic extractants/solvents or pass through a chromatographic column, making these separations impractical. In fact, both Am(VI) and Am(V) are traditionally thought to be unstable in aqueous solution because they can even be efficiently reduced by active radiolysis products, given that the two common americium isotopes related to the nuclear fuel cycle (^241^Am and ^243^Am) are both considerably radioactive.

**Fig. 1:**
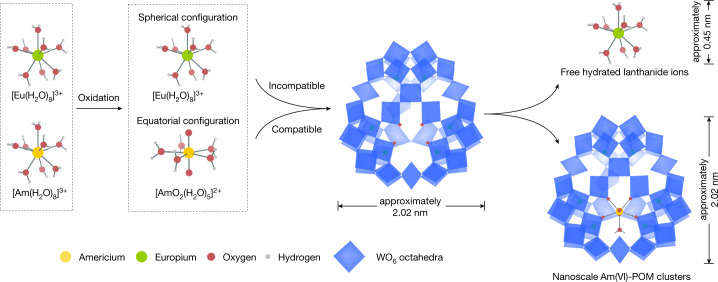
Schematic illustration of the frame of the ultrafiltration separation of nanoscale Am(VI)-POM clusters from lanthanides. Am(III) is first oxidized to Am(VI), which can be selectively coordinated and stabilized by a POM cluster with a vacant equatorial donor site. This POM is unsuitable for binding Ln(III), thereby leading to a large size difference between Am(VI)-POM clusters and Ln(III) ions, which is then used as a basis for their ultrafiltration separation.

We address these challenges by selecting a polyoxometalate (POM) that is tailored to the coordination requirements of Am(VI) and discriminates against Ln(III) cations. POMs are a well-known class of nanoscale, inorganic, metal-oxo clusters assembled from MO_x_ units (M = V, Mo, W; x = 4–6), whose coordination chemistry with transuranium elements have seldom been investigated^14–16^. This POM is equipped with a vacant equatorial donor site precisely matching the common pentagonal bipyramid coordination geometry of an actinyl ion and is unsuitable for binding Ln(III) ions. Such precise and strong coordination by a large cluster not only stabilizes Am(VI) to an unmatched level but also efficiently discriminates americium and lanthanides with a large size difference between their coexisting chemical species (Fig. 1). When combined with an industrial ultrafiltration technique, these efforts give rise to a new separation method.

The lacunary POM {Se_6_W_45_} was synthesized through self-assembly of the {Se_6_W_39_} precursor^17,18^ in acid solution at 300 K (detailed synthetic procedures can be found in Methods). The initial {Se_6_W_39_} possesses a macrocyclic structure with a cavity size of 8.7 × 8.7 Å. After self-assembly, the cavity on the front of the POM is plugged by three WO_6_^6−^ groups, and the back of the POM is capped by three further WO_6_^6−^ groups, leaving a vacancy site with preorganized coplanar oxo-donor structure for binding actinyl ions (Fig. 1 and Supplementary Fig. 1), as demonstrated by single-crystal X-ray diffraction analysis. We observed that {Se_6_W_45_} is prone to forming nanoscale clusters in nitric acid. As shown in Supplementary Fig. 2, dynamic light scattering measurements indicate that the hydrodynamic diameter of {Se_6_W_45_} is approximately 2.6 nm, consistent with the crystallographic result in the dimension of 2.02 × 2.02 × 1.02 nm. Raman spectra recorded from solutions of {Se_6_W_45_} stored in 0.1 M of nitric acid for 1 week showed that it has sufficient stability to be used in these harsh conditions (Supplementary Fig. 3).

**Fig. 2:**
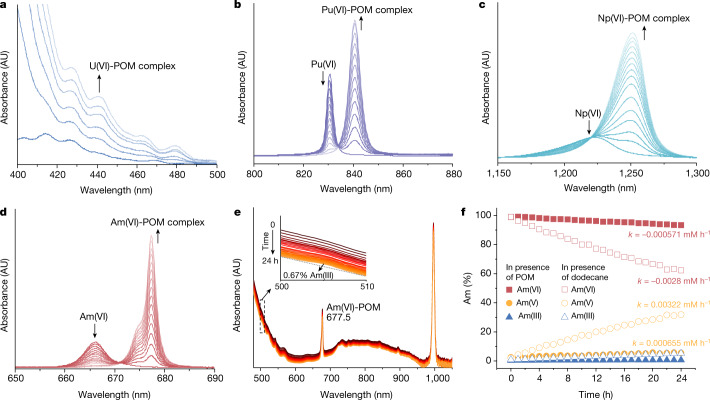
Absorption spectra of An(VI)-POM in aqueous solution. **a**–**d**, Spectrophotometric titrations of POM complexing with hexavalent actinyl ions in 0.1 M of HNO_3_ aqueous solution: UO_2_^2+^ (**a**), PuO_2_^2+^ (**b**), NpO_2_^2+^ (**c**) and AmO_2_^2+^ (**d**). **e**, Change in the absorption spectra of the aqueous solution containing 0.25 mM of Am in 0.1 M of HNO_3_, which was oxidized with Cu(III) periodate, in the presence of 2.0 eq. POM over a period of 24 h. **f**, Reduction kinetics of Am(VI)-POM in 0.1 M of HNO_3_. AU, arbitrary unit.

To investigate the complexation properties of actinyl ions with the POM cluster in nitric acid, we monitored the change in the absorption spectra of AnO_2_^2+^ ions (An = ^238^U, ^237^Np, ^242^Pu or ^243^Am) with the addition of the {Se_6_W_45_} POM. The absorption spectrum of UO_2_^2+^–{Se_6_W_45_} shows the typical charge transfer of the uranyl cation as shown in Fig. 2a. The characteristic absorption bands of NpO_2_^2+^, PuO_2_^2+^ and AmO_2_^2+^ originating from Laporte-forbidden 5*f*→5*f* transitions experience notable bathochromic shifts on POM addition (Np(VI) from 1224 nm to 1251 nm, Pu(VI) from 831 nm to 841 nm and Am(VI) from 666 nm to 677 nm) (Fig. 2b–d), initially implying strong complexation between AnO_2_^2+^ ions and the POM^19^. In particular, we performed a spectrophotometric titration of the AmO_2_^2+^–{Se_6_W_45_} system to gain quantitative insight into the complexation process. The characteristic band of free AmO_2_^2+^ ions at 666 nm gradually decreases, and a new band at 677 nm emerges concurrently during the titration (Fig. 2d). Fitting these titration data suggests the formation of a 1:1 AmO_2_^2+^/{Se_6_W_45_} complex, and its formation constant (log *β*) was calculated as 6.17 ± 0.10 (Supplementary Fig. 4). This value confirms strong complexation between hexavalent actinyl ions and POM in 0.1 M of nitric acid (Supplementary Table 1). By sharp contrast, the results of spectrophotometric titration of Nd(III) with {Se_6_W_45_} and luminescence lifetime titration of Eu(III) with {Se_6_W_45_} imply that the Ln/POM complex with a stoichiometry of 1:2 formed in the solution mainly by weak electrostatic interactions between the hydrated Ln ions and the negatively charged POM anions, instead of inner-sphere coordination (Supplementary Figs. 5 and 6 and Supplementary Table 2). These observations are consistent with the preliminary results of the ab initio molecular dynamics (AIMD) simulations, which suggest that the vacancy site in the POM is much more suitable for binding actinyl(VI) over trivalent lanthanides (Supplementary Videos 1–4). More notably, the strong complexation stabilizes ^243^Am(VI), and only 0.67% of the Am(VI) was reduced in the presence of POM clusters over a period of 24 h (Fig. 2e) by radiolysis products. In addition, the reduction kinetics rate of Am(VI) in the POM system is −5.71 × 10^−4^ mM h^−1^, which is significantly slower than the rates of other investigated systems (Fig. 2f)^6,7,20,21^. Investigation of the POM and the Am(VI)-POM adduct by electrochemistry in nitric acid solutions confirms the substantial stabilization of the Am(VI) by the POM (E_p/2,Ox_(Am(VI)) = 1.33 V versus SCE and E_p/2,Ox_(Am(VI)-POM) = 1.15 V versus SCE) and a significant reduction in the oxidizing power of Am(VI) while coordinated to the POM (E_p/2,Red_(Am(VI)) = 1.18 V versus SCE and E_p/2,Red_(Am(VI)-POM) = 0.21 V versus SCE; Supplementary Figs. 7–11 and Supplementary Table 3), which is highly consistent with the notably slower reduction kinetics observed for the Am(VI)-POM adduct. The solution chemistry investigation demonstrates that Am(VI) could persist to a previously unachievable level for separation applications with the aid of strong complexation by inorganic POM clusters.

**Fig. 3:**
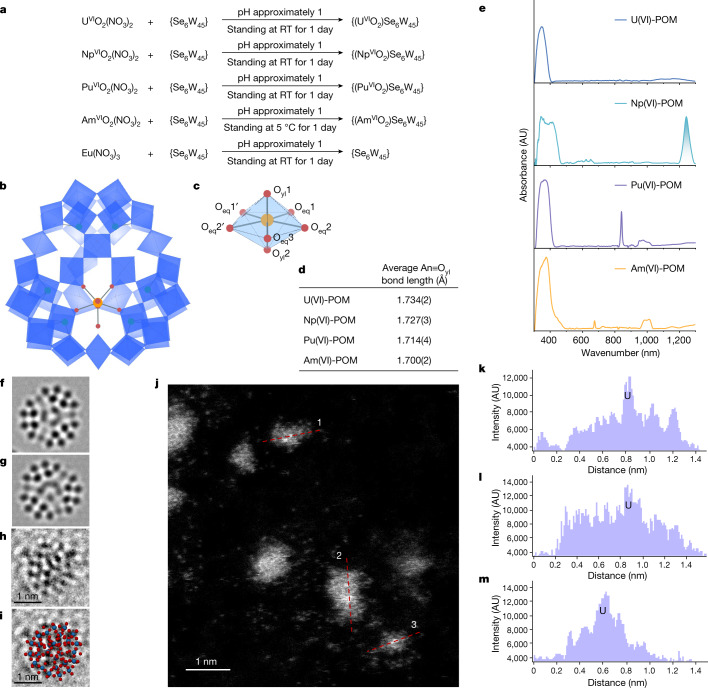
Graphical representation of the structures of An-POM and absorption spectra of their single crystals. **a**, Chemical equations of POM assembled reactions with actinides or lanthanides. **b**, Polyhedral and ball-and-stick representation of Am(VI)-POM, with the H atoms and counterions omitted for clarity. Colour code: blue octahedra, WO_6_; orange ball, Am; red balls, O; green balls, Se. **c**, The pentagonal bipyramid coordination geometry of Am(VI). **d**, The table of average An≡O bond length. **e**, UV–vis–NIR spectra of single crystal U(VI)-POM, Np(VI)-POM, Pu(VI)-POM, and Am(VI)-POM. **f**,**g**, ACTEM imaging simulations of POM (**f**) and U(VI)-POM (**g**) on graphene substrate. **h**,**i**, Raw ACTEM images of a POM (**h**) and with POM molecular model overlapped (**i**). **j**, Raw HAADF STEM image of U(VI)-POM. **k**,**l**,**m**, Intensity profiles of the lines 1 (**k**), 2 (**l**) and 3 (**m**) in **j**, showing the existence and position of U ions in U(VI)-POM. RT, room temperature.

To directly visualize the interaction between AnO_2_^2+^ ions and the POM cluster, we prepared a series of actinyl-POM crystals by reacting AnO_2_^2+^ ions (An = ^238^U, ^237^Np, ^242^Pu or ^243^Am) with {Se_6_W_45_} in solution (Fig. 3a and Supplementary Fig. 12). Single-crystal X-ray diffraction analysis shows that actinyl-POMs from U to Am are isomorphous and crystallize in the monoclinic space group *P*2_1_/*m*. The actinyl ions are fully encapsulated within the predesigned vacant site. The equatorial oxygen atoms of actinyl ions are provided by four distinct WO_6_^6−^ groups and one coordinated water, forming a pentagonal bipyramid coordination geometry (Fig. 3b,c). To demonstrate the oxidation state of actinyl ions in the POM cluster, solid-state ultraviolet–visible–near-infrared (UV–vis–NIR) absorption spectra were recorded on these single crystals. Figure 3e shows the typical electronic transitions associated with *f*-elements in the hexavalent state, including charge-transfer transitions at 349 nm (UO_2_^2+^) and 5*f*→5*f* transitions at 841 nm (PuO_2_^2+^), 1242 nm (NpO_2_^2+^) and 674 nm (AmO_2_^2+^). In addition, crystallographic investigation confirms that the actinide contraction effect dominates the An≡O axial bond distances and An-O equatorial bond distances. In the actinyl-POM cluster, the average An≡O axial bond distances are 1.734(2) Å, 1.727(6) Å, 1.714(4) Å and 1.700(2) Å for U(VI), Np(VI), Pu(VI) and Am(VI), respectively (Fig. 3d and Supplementary Table 4), where the number in parentheses is the uncertainty value for bond length. Note that the An≡O axial bond distances in the POM are approximately 0.03 Å shorter than the bond distances in other oxoanion and organometallic compounds^22^. The different charge distributions in the interior and surface of the POM tend to polarize the O≡An≡O axial bonds, thus leading to elongation of one bond and shortening of the other when the actinyl ion is vertically encapsulated within the vacancy site^23–25^. By sharp contrast, when Eu^3+^ ions, a representative Ln(III) ion, react with the POM under the same conditions, only pure {Se_6_W_45_} crystals are isolated without the presence of any lanthanide ions in the structure. In addition, we confirmed the atomic structure of a single particle of a POM and a U-POM cluster by combined aberration-corrected transmission electron microscopy (ACTEM), high-angle annular dark-field scanning transmission electron microscopy (HAADF STEM) and transmission electron microscopy (TEM) imaging simulations (Fig. 3f–m and Supplementary Figs. 13–15). The solo U ion is observed located in the near centre of the POM cluster (Fig. 3g,j–m). Energy-dispersive spectrometry mapping also confirms their elemental compositions, as shown in Supplementary Fig. 15.

A comparison of the binding energies for An(VI)-POM complexes (−508.26 to −491.22 kcal mol^−1^) probed by density functional theory (DFT) calculations shows that they are significantly larger than the values for complex formation of lanthanides with the POM cluster (−37.50 kcal mol^−1^ for Nd^3+^ and −37.74 kcal mol^−1^ for Eu^3+^) because of pronounced electrostatic attraction and orbital interactions of actinyl ions with POM clusters when compared with lanthanides (Supplementary Table 5 and Supplementary Figs. 16–18). Overall, the combination of crystallographic results, spectroscopy data, TEM data and computation results corroborate that the vacancy site in {Se_6_W_45_} POM precisely matches the coordination geometry of actinyl(VI) ions and is unsuitable for binding Ln(III) ions.

**Fig. 4:**
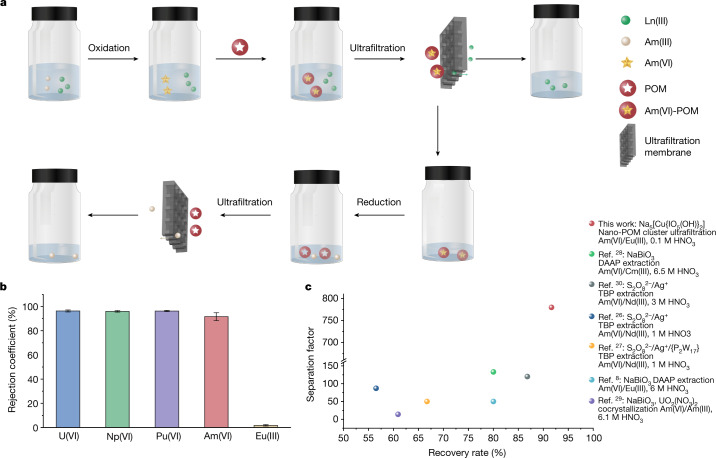
Demonstration of Am separation through oxidation, complexing and ultrafiltration using POMs as complexants. **a**, Depiction of actinide group separation strategies. **b**, Ultrafiltration separation results of U(VI), Np(VI), Pu(VI), Am(VI) and Eu(III). The experimental condition: initial An(VI) or Eu(III) concentration is about 2.0 × 10^−5^ mol l^−1^, POM concentration is about 4.0 × 10^−5^ mol l^−1^, HNO_3_ concentration is 0.1 mol l^−1^, NH_4_NO_3_ concentration is 0.15 mol l^−1^ and the reaction time is 5 min. **c**, Comparisons with the representative Am(VI)-based separation performances on the recovery rate of Am and separation factor. DAAP, diamylamylphosphonate; TBP, tributyl phosphate.

Owing to size and charge differences for Am(VI)-POM clusters and hydrated lanthanide ions in nitric acid solution, we designed a separation protocol relying on a commercially available ultrafiltration technique. The whole separation procedure, using the oxidization of the actinides, nanoscale cluster assembly and ultrafiltration separation, can be accomplished homogeneously in minutes using the aqueous solution with no organic component involved (Fig. 4a). This significantly reduces the amount of secondary radioactive waste. The purified Am(VI)-POM can be further reduced to obtain Am(III) products, and the released POM clusters can be recyclable again by ultrafiltration for the next separation cycle (Fig. 4a). After the screening of condition parameters such as acidity, reaction time and the type and concentration of counterions (Supplementary Figs. 19–21), we tested the optimized recovery of AnO_2_^2+^ ions (An = ^238^U, ^237^Np, ^242^Pu or ^243^Am) after the ultrafiltration process. As shown in Fig. 4b and Supplementary Fig. 22, the rejection coefficients of actinyl ions are higher than 96% for U (96.33% ± 0.79%), Np (96.00% ± 0.62%) and Pu (96.28% ± 0.50%), and are 91% for Am (91.64% ± 3.23%). The rejection coefficient of Eu(III) is low at 1.73% ± 0.58%. These results give rise to an Am(VI)/Eu(III) separation factor of 780 that is significantly higher than those of reported Am(VI) oxidation state associated separation techniques^8,26–32^ (Fig. 4c).

The foregoing results demonstrate that control of the lacunary structure in a POM cluster enables the unprecedented complexation and stabilization of Am(VI) in aqueous solution, leading to a new separation strategy with merits of high efficiency, absence of organic components, low time cost and low energy input. This idea also opens a new opportunity for actinide group separation from fission products during the reprocessing of used nuclear fuel.

## Methods

### General considerations

Cautions! The related isotopes of Np, Pu and Am exhibit significant radio- and chemo-toxicity and represent serious health risks when inhaled or digested. ^237^Np (half-life (*t*_1/2_) = 2.14 × 10^6^ years, specific activity = 0.7 mCi g^−1^) is a strong α emitter, and decays to the short-lived isotope ^233^Pa (*t*_1/2_ = 27.0 days), which is a potent β and γ emitter. ^242^Pu (*t*_1/2_ = 3.76 × 10^5^ years, specific activity = 3.9 mCi g^−1^) is a strong α emitter. ^243^Am (*t*_1/2_ = 7,380 years, specific activity = 199 mCi g^−1^) is a strong α emitter with γ emission, presenting internal and external radiotoxic hazards. All experimental studies were conducted in licensed laboratories dedicated to transuranium elements study with approved safety operating procedures. All neptunium, plutonium and americium materials have to be handled in either negative-pressure radiological fume hoods or glove boxes equipped with high-efficiency particulate air filters, and high-level precautions and procedures for handling radioactive materials must be followed.

### Synthesis

All reagents were purchased from chemical reagent suppliers and used without further purification. Millipore water was used in all experiments. The POM precursor of {Se_6_W_39_} was synthesized according to the previously reported procedure^17,18^. ^237^NpO_2_ powder was obtained from the China Institute of Atomic Energy. ^242^PuO_2_ powder and ^243^AmO_2_ powder were purchased from the Shenzhen Isotope Industrial International Co., Ltd, and were originally produced in Los Alamos National Laboratory, USA (sample identification of No. Pu-242-237-A and Am-1-91-Prod). In the ^242^PuO_2_ sample, the weight ratios of ^242^Pu, ^238^Pu, ^239^Pu, ^240^Pu, ^241^Pu and ^244^Pu were 99.9628%, 0.0029%, 0.0049%, 0.0217%, 0.0056% and 0.0020%, respectively. In the ^243^AmO_2_ sample, the weight ratios of ^243^Am, ^241^Am and ^242^Am were 99.987%, 0.012% and less than 0.001%, respectively. The oxidation of Am(III) to Am(VI) in aqueous solution was achieved by Cu(III) periodate oxidant according to the reported procedure^20^.

#### Preparation of ^237^NpO_2_^2+^ stock solution

We obtained 0.04 M of stock solution of NpO_2_^+^ (perchlorate) from dissolution of NpO_2_. Heating the NpO_2_^+^ solution with the addition of concentrated HNO_3_ affords NpO_2_^2+^ residues. In the heating process, a large excess of NO_2_, decomposed from HNO_3_, fully oxidizes Np(V) to Np(VI). Then, 0.1 M of Np^VI^O_2_^2+^ stock solution was obtained in a pink colour with the addition of 0.1 M of HNO_3_, as shown in Supplementary Fig. 23. The oxidation state of Np was probed by UV–vis–NIR spectrometry, with the disappearance of the characteristic absorption peak at 980 nm (Np(V)) and emergence of the characteristic absorption peak of 1224 nm for Np(VI).

#### Preparation of ^242^PuO_2_^2+^ stock solution

The stock solution of PuO_2_^2+^ in nitric acid was prepared by repeatedly dissolving PuO_2_ powder in concentrated nitric acid in an autoclave at 473 K. Greenish Pu(NO_3_)_4_ solutions were obtained, and followed by distillation in a sand bath at ambient pressure. During this process, HNO_3_ decomposes to NO_2_, which further oxidizes Pu(IV) to Pu(VI). Then, diluted HNO_3_ was added to the residues, obtaining a 0.1 M ^242^PuO_2_^2+^ stock solution (Supplementary Fig. 24).

#### Preparation of ^243^AmO_2_^2+^ solution

We obtained 0.01 M of Am(III) solution by dissolving AmO_2_ powder in 0.1 M of nitric acid in a glass vial at room temperature for 3 days. 0.01 M AmO_2_^2+^ solution was prepared by the addition of Cu(III) periodate into the solution of Am(III), in which approximately 99.9% of the Am(III) was oxidized to Am(VI), monitored by UV–vis–NIR spectroscopy. The solution of AmO_2_^2+^ was used promptly in the following experiments once in preparation (Supplementary Fig. 25).

#### Synthesis of [(CH_3_)_2_NH_2_]_15_Na_0.8_(H_3_O)_8.2_[Se_6_W_45_O_159_(H_2_O)_9_]·27H_2_O ({Se_6_W_45_})

We dissolved 150 mg of {Se_6_W_39_} in 2 ml of water, and then added 2 ml of (CH_3_)_2_NH_2_Cl aqueous solution (75 mg ml^−1^), 1 ml of water and 0.2 ml of HNO_3_ (8 mol l^−1^). Colourless crystals were obtained at 300 K after 1 day. The yield was 0.074 g (46% based on W). For Se_6_W_45_Na_0.8_O_203.2_C_30_N_15_H_216.6_, the calculated values were as follows: C, 2.81%; H, 1.70%; N, 1.64%; Na, 0.14%; Se, 3.69%; and W, 64.60%. The values we found were as follows: C, 3.18%; H, 1.34%; N, 1.68%; Na, 0.14%; Se, 3.73%; and W, 63.29%. The lattice water was calculated by thermogravimetric analysis (TGA), as shown in Supplementary Fig. 26. The coordinated water was determined by bond valence sum (BVS) calculation (Supplementary Table 6). The dimethyl ammonium cations were determined by CHN elemental analysis (Supplementary Table 11). The amount of Na was determined by inductively coupled plasma mass spectrometry (ICP-MS). The amounts of Se and W were determined by inductively coupled plasma optical emission spectrometry (ICP-OES) and energy-dispersive X-ray spectroscopy (EDS), as shown in Supplementary Fig. 27.

#### Synthesis of [(CH_3_)_2_NH_2_]_15_(H_3_O)_7_[(UO_2_)Se_6_W_45_O_159_(H_2_O)_10_]·24H_2_O (U(VI)-POM)

We dissolved 170 mg of {Se_6_W_45_} in 2 ml of HNO_3_ solution (0.1 mol l^−1^), and then added 2.0 ml of HNO_3_ solution (0.1 mol l^−1^) containing 150 mg of (CH_3_)_2_NH_2_Cl. After that, 1.0 ml of HNO_3_ solution (0.1 mol l^−1^) containing UO_2_(NO_3_)_2_∙6H_2_O (5.5 mg, 0.011 mmol) was added slowly to the prepared POM solution with stirring. Yellow rod-shaped crystals of U(VI)-POM were isolated at 300 K after 1 day. For USe_6_W_45_O_202_C_30_N_15_H_209_, the calculated values were as follows: C, 2.77%; H, 1.83%; N, 1.61%; Se, 3.64%; and W, 63.58%. The values we found were as follows: C, 3.37%; H, 1.41%; N, 1.60%; Se, 3.96%; and W, 63.02%. The lattice water was calculated by TGA, as shown in Supplementary Fig. 28a. The coordinated water was determined by BVS calculation (Supplementary Table 7). The dimethyl ammonium cations were determined by CHN elemental analysis (Supplementary Table 11). The amount of Na determined by ICP-MS was low at 0.062%. The amounts of Se, W and U were determined by ICP-OES.

#### Synthesis of [(CH_3_)_2_NH_2_]_x_(H_3_O)_22-x_[(NpO_2_)Se_6_W_45_O_159_(H_2_O)_10_]·nH_2_O (Np(VI)-POM)

We dissolved 35 mg of {Se_6_W_45_} in 400 μl of HNO_3_ solution (0.1 mol l^−1^), and then added 400 μl of HNO_3_ solution (0.1 mol l^−1^) containing 30 mg of (CH_3_)_2_NH_2_Cl. After that, a stock solution of Np^VI^O_2_^2+^ nitrate containing 1.0 mg Np(VI) was slowly added to the prepared POM solution. Light-yellow rod-shaped crystals of Np(VI)-POM were isolated at 300 K after 1 day. The coordinated water was determined by BVS calculation (Supplementary Table 8).

#### Synthesis of [(CH_3_)_2_NH_2_]_x_(H_3_O)_22-x_[(PuO_2_)Se_6_W_45_O_159_(H_2_O)_10_]·nH_2_O (Pu(VI)-POM)

We dissolved 35 mg of {Se_6_W_45_} in 400 μl of HNO_3_ solution (0.1 mol l^−1^), and then added 400 μl of HNO_3_ solution (0.1 mol l^−1^) containing 30 mg of (CH_3_)_2_NH_2_Cl. After that, a stock solution of PuO_2_^2+^ nitrate containing 1.0 mg of Pu(VI) was slowly loaded into the prepared POM solution. Light yellow rod-shaped crystals of Pu(VI)-POM were isolated at 300 K after 1 day. The coordinated water was determined by BVS calculation (Supplementary Table 9).

#### Synthesis of K_12_[(CH_3_)_2_NH_2_]_4_(H_3_O)_6_[(UO_2_)Se_6_W_45_O_159_ (H_2_O)_10_]·32H_2_O (U(VI)-POM-2)

We dissolved 3.5 mg of {Se_6_W_45_} in 40 μl of HNO_3_ solution (0.1 mol l^−1^), and then added 100 μl of HNO_3_ solution (0.1 mol l^−1^) containing UO_2_(NO_3_)_2_∙6H_2_O (3 mM). After that, 30 μl of KNO_3_ solution (2.5 mol l^−1^) was added to the solution. The mixed solution was stored in a fridge at 277 K. Light yellow block crystals were isolated after 6 h. For USe_6_W_45_K_12_C_8_H_134_N_4_O_209_, the calculated values were as follows: C, 0.73%; H, 1.03%; N, 0.43%; Se, 3.62%; W, 63.22%; and U, 1.81%. The values we found were as follows: C, 0.76%; H, 0.99%; N, 0.72%; Se, 3.56%; W, 64.35%; and U, 1.75%. The lattice water was calculated by TGA (Supplementary Fig. 28b). The dimethyl ammonium cations were determined by CHN elemental analysis (Supplementary Table 11). The amount of Na determined by ICP-MS was 0.047%, which is negligible. The amounts of Se, W, U and K were determined by ICP-OES.

#### Synthesis of K_x_[(CH_3_)_2_NH_2_]_y_(H_3_O)_22-x-y_[(AmO_2_)Se_6_W_45_O_159_ (H_2_O)_10_]·nH_2_O (Am(VI)-POM)

Addition of 2.5 mg Cu(III) periodate to the 100 μl Am(III) solution (0.6 mg Am in mass) generated an Am(VI) solution. Then, 3.5 mg of {Se_6_W_45_}, dissolved in 40 μl of HNO_3_ solution (0.1 mol l^−1^), was added. After that, 30 μl of KNO_3_ solution (2.5 mol l^−1^) was added to the solution. The mixed solution was stored in a fridge at 277 K. Golden-yellow block crystals were isolated after 6 h. The ratio of Am to POM in the Am(VI)-POM complex was close to 1:1, which was determined by liquid scintillation counting and Uv–vis spectroscopy data (Supplementary Fig. 29). The coordinated water was determined by BVS calculation (Supplementary Table 10).

The synthetic procedures of An(VI)-POM crystals are summarized in Supplementary Fig. 30.

### Elemental analysis

The amounts of Se, W, U, K and Na were determined on an iCAP 7200 ICP-OES Radial (Thermo Fisher Scientific) and ELEMENT 2 ICP-MS (Thermo Fisher Scientific). The EDS analysis was carried out on a Regulus SU8230 Field Emission SEM (Hitachi). The CHN content microanalysis to determine CHN content was performed on a UNICUBE elemental analyzer (Elementar).

### UV–vis–NIR spectrophotometric titration

The UV–vis–NIR spectra were collected with a Cary 6000i spectrophotometer (Agilent) using quartz cuvettes with a 1 cm path length, with the configuration of bandwidth of 0.5 nm and an integration time of 0.10 s. Typically, 0.400 ml of Am(VI) (0.575 mM) in a 1.0 cm quartz cell was titrated with the POM solution (2.0 mM, 10 μl per addition) through a 10 μl pipette. After each addition, the solutions were electromagnetically stirred for 5 min before recording the spectra. Preliminary kinetic experiments demonstrated that the reaction reaches equilibrium in less than 5 min. The formation constants were then calculated by non-linear regression using the program of HypSpec^33^ based on the obtained spectral data. The titration procedure for Pu(VI), Np(VI) and U(VI) is similar to that for Am(VI); the concentrations of Pu(VI), Np(VI) and U(VI) are 0.1 mM, 0.45 mM and 0.1 mM, respectively.

The stability constant (log *β*) of the complex, also known as the formation constant or the binding constant, is the equilibrium constant of the complex formed in the solution, which can represent the strength of the binding of the metal ion and the complexing agent.

The formation of Am(VI)-POM can be described by the equation:

1 $${\rm{Am}}({\rm{VI}})+{\rm{POM}}\rightleftharpoons {\rm{Am}}({\rm{VI}})-{\rm{POM}}.$$

The conditional stability constant of the complex, log *β*, is given by the following equation:

2 $$\log \beta =\log \frac{[{\rm{A}}{\rm{m}}({\rm{V}}{\rm{I}})-{\rm{P}}{\rm{O}}{\rm{M}}]}{[{\rm{A}}{\rm{m}}({\rm{V}}{\rm{I}})]\times [{\rm{P}}{\rm{O}}{\rm{M}}]},$$

where [Am(VI)], [POM] and [Am(VI)-POM] represent the concentrations of Am(VI), POM and Am(VI)-POM in the solution, respectively. Spectrophotometric technique can be used to determine the concentrations of Am(VI) and Am(VI)-POM in a solution, and the stability constants could be calculated according to equation (2).

### Solid-state UV–vis–NIR spectroscopy

Solid-state UV–vis–NIR spectra were recorded using a CRAIC Technologies microspectrophotometer. Crystals (except for Am(VI)-POM) were placed on a quartz slide under immersion oil and the data were collected from 400 nm to 1,300 nm at 300 K. Because Am(VI) in the crystal of Am(VI)-POM can be facilely reduced, contact with oil should be avoided.

### Crystallographic studies

Single-crystal X-ray diffraction data were collected on a Bruker D8 Venture diffractometer with a Turbo X-ray Source (Mo-Kα radiation, *λ* = 0.71073 Å), adopting the direct-drive rotating anode technique and a Complementary Metal Oxide Semiconductor (CMOS) detector at room temperature. The data frames were collected using the program APEX3 and processed using the SAINT routine in APEX3. Using Olex2^34^, the structure was solved by the ShelXT^35^ structure solution program using Intrinsic Phasing and refined with the ShelXL^36^ refinement package using least squares minimization. The solvate molecules and counterions of all data were treated as a diffuse contribution to the overall scattering without specific atom positions by SQUEEZE/PLATON^37^ because of their severe disorder in the lattices. The details on the treatment of the crystallographic disorder of W atoms in POM and An(VI)-POM are provided in Supplementary Figs. 31–40. The crystallographic data of POM and An(VI)-POM are shown in Supplementary Table 12.

All structures were solved by direct method and refined by full-matrix least squares on *F*^2^. In the crystal structure of the POM, the positional disorder over three positions was observed for three W atoms around the hole of the POM. The initial site occupancy factor of W atoms was set to free variables. The refinement gives a site occupancy factor of 0.687 for W21, W22 and W21’, respectively. Then, the configurations 2 and 3 were resolved accordingly. The crystal structures of An(VI)-POM were solved by the same procedure. The partially occupied actinide atoms on each side of the structure were separated into different parts using RESI and PART instructions with reasonable distance and connection. The free variable of the disorder ratio was set to the same to guarantee the full occupancy of the actinide atom. The missing O atoms with low occupancy were modelled in reference to the model of the other side of the structure, as well as the coordination environment of W and actinide atoms. The DFIX command was used to constrain the geometry to make the sites more reasonable. Some difficult sites were found using FRAG and FEND instructions. The SAME command was used to constrain the geometry of similar parts to the ‘clearer one’. After modelling all atoms, anisotropic refinement was performed together with the SIMU command.

### Computational details

All the theoretical calculations were carried out at the level of DFT using the Amsterdam Density Functional program (ADF 2019.301)^38^. The theoretical results were performed in the generalized gradient approximation with the Perdew–Burke–Ernzerhof (PBE) exchange-correlation functional^39^. The zero-order regular approximation (ZORA)^40^ was adopted to account for the scalar relativistic effects. The TZ2P (containing valence triple zeta and two polarization functions), TZP (containing valence triple zeta and one polarization function) and DZP (containing valence double zeta and one polarization function) basis sets were used as follows: TZ2P for U, Np, Pu, Am, Nd and Eu; TZP for W; and DZP for H, O and Se^41^. The frozen core approximation was used as [1s^2^-4f^14^] for U, Np, Pu, Am and W; as [1s^2^-4d^10^] for Nd and Eu; as [1s^2^-3d^10^] for Se; and as [1s^2^] for O. The conductor-like screening model^42^ was also used with the water environment. The energy decomposition analysis with natural orbitals for chemical valence (EDA-NOCV)^43,44^ was calculated to analyse the chemical bonding properties using the ADF 2019.301 program. The AIMD simulations with partial geometric constraints of Nd(III)–POM and U(VI)-POM were performed using DFT with PBE^39^ exchange-correlation functional as implemented in the CP2K package^45,46^. The core electrons have been modelled by scalar relativistic norm-conserving pseudopotentials^47^ with 6, 6, 14, 14 and 14 valence electrons of O, Se, W, Nd and U, respectively^48,49^. All AIMD simulations were done in the NVT (number of particles, absolute temperature, volume) ensemble in 25.00-Å cubic boxes. The Nosé–Hoover thermostat was used with a step of 0.5 fs at 300 K (refs. ^50,51^).

### Electron microscopy studies

All the materials were dispersed in methanol and drop-cast onto graphene-coated copper TEM grids. Aberration-corrected high-resolution TEM images, HAADF STEM images and EDS mapping were carried out on double Cs-corrected JEOL GRAND ARM 300F with a cold field-emission gun operated at 80 kV at room temperature. The TEM specimen was heated in air at 423 K for 5 min shortly before insertion into the TEM column. TEM image simulation was carried out using the multislice program QSTEM.

### Separation experiments

The ultrafiltration separation of UO_2_^2+^/Eu^3+^ was conducted at 300 K. The pH was adjusted to 1.0 by adding a small volume of concentrated HNO_3_. The solution of POM was added in the solution of UO_2_^2+^/Eu^3+^, then the solution of NH_4_NO_3_ was added. The mixed solution was shaken several times on a shaker and transferred to a centrifugal concentrator (modified polyethersulfone ultrafiltration membrane (Pall) with 3-kDa molecular weight cut-off). They were centrifuged to separate the An-POM complexes from lanthanide ions. The concentrations of U and Eu in the initial solution and final filtrate were determined by ICP-AES and ICP-MS, respectively. The rejection coefficient (*R*) and the separation factor (SF) were calculated from the following equation:

3 $$R=\left(1-\frac{{c}_{{\rm{p}}}}{{c}_{{\rm{f}}}}\right)\times 100 \% ,$$

4 $${{\rm{SF}}}_{{\rm{U/Eu}}}={\left(\frac{{c}_{{\rm{f}}}-{c}_{{\rm{p}}}}{{c}_{{\rm{p}}}}\right)}_{{\rm{U}}}\,/{\left(\frac{{c}_{{\rm{f}}}-{c}_{{\rm{p}}}}{{c}_{{\rm{p}}}}\right)}_{{\rm{Eu}}},$$

where *c*_f_ and *c*_p_ are the concentration of metal ion in the feed and permeate streams, respectively.

The ultrafiltration separations of Pu(VI), Np(VI) and Am(VI) by POM were conducted at 300 K. The solution of POM in 0.1 M of HNO_3_ solution was added to the solution of Pu(VI), Np(VI) and Am(VI). Then, the solution of NH_4_NO_3_ was added. The mixed solution was shaken several times on a shaker at 300 K and transferred to a centrifugal concentrator (modified polyethersulfone ultrafiltration membrane (Pall) with 3-kDa molecular weight cut-off). The systems were centrifuged to retain the An-POM complexes. The amount of An(VI) was determined by taking an aliquot of 0.100 ml of the initial solution and the filtrate and mixing it with 10 ml of a scintillation cocktail for liquid scintillation counting on an ultra-low background liquid-scintillation spectrometer (Quantulus 1220, PerkinElmer). The rejection coefficient (*R*) for a given radioisotope was calculated from the following equation:

5 $$R=\left(1-\frac{{C}_{{\rm{p}}}}{{C}_{{\rm{f}}}}\right)\times 100 \% $$

where *C*_f_ and *C*_p_ are the counting rates (counts per minute) of the radioactive nuclides per unit volume in the feed and permeate streams, respectively.

To quantify the separation between An(VI) and Eu(III), the theoretical separation factor (SF) was calculated by the formula:

6 $${{\rm{SF}}}_{{\rm{An}}/{\rm{Eu}}}={\left(\frac{{c}_{{\rm{f}}}-{c}_{{\rm{p}}}}{{{\rm{c}}}_{{\rm{p}}}}\right)}_{{\rm{An}}}\,/{\left(\frac{{{\rm{c}}}_{{\rm{f}}}-{{\rm{c}}}_{{\rm{p}}}}{{{\rm{c}}}_{{\rm{p}}}}\right)}_{{\rm{Eu}}}$$

where *c*_f_ and *c*_p_ are the are the concentration of metal ion in the feed and permeate streams, respectively. The concentrations of Np, Pu and Am were determined by liquid scintillation. The concentration of Eu was determined by inductively coupled plasma analysis.

### Other physical characterizations

The Raman spectra of the POM {Se_6_W_45_} in the solid state and in aqueous solutions were recorded using a confocal LabRAM HR800 spectrometer (HORIBA Jobin Yvon). A laser with an excitation wavelength of 633 nm was used for the measurements in the solid state. A laser with an excitation wavelength of 514 nm was used for the measurements in aqueous solutions. Elemental analysis (C, H and N) was performed on a Vario EL CHNOS elemental analyzer. TGA was carried out on a NETZSCH STA 449F3 instrument in the range of 30–800 °C under a nitrogen flow at a heating rate of 10 °C min^−1^. The dynamic light scattering was measured with a Zetasizer Nano ZS90 analyzer. The concertation of each element in separation experiments was determined by ICP-MS for U, by ICP-OES for Eu and by Quantulus 1220 liquid scintillation counter (PerkinElmer) for isotopes of Np, Pu and Am. The γ-ray energy spectrum of the ^243^Am solution was tested by a NaI scintillator detector with a standard ORTEC NIM modules. The spectrum was then generated by the MAESTRO-32 software. Electrochemical tests were carried out on a CHI 760E electrochemical workstation at 300 K. A conventional three-electrode system was used with a 3 mm glassy carbon electrode as the working electrode, an SCE as the reference electrode and a platinum wire as the counter electrode.

## Online content

Any methods, additional references, Nature Portfolio reporting summaries, source data, extended data, supplementary information, acknowledgements, peer review information; details of author contributions and competing interests; and statements of data and code availability are available at 10.1038/s41586-023-05840-z.

## Supplementary information

Supplementary Information Supplementary Sections 1–22, including Figs. 1–40, Tables 1–12 and References.

Supplementary Video 1 The progress of AIMD simulations of the U(VI)-POM system (top view).

Supplementary Video 2 The progress of AIMD simulations of the U(VI)-POM system (side view).

Supplementary Video 3 The progress of AIMD simulations of the Nd(III)-POM system (top view).

Supplementary Video 4 The progress of AIMD simulations of the Nd(III)-POM system (side view).

Supplementary Data Supplementary data cif files with CCDC deposit numbers from 2150690 to 2150694. These can be obtained free of charge from The Cambridge Crystallographic Data Centre via http://www.ccdc.cam.ac.uk/data_request/cif.

## Data Availability

The data that support the findings of this study can be obtained from the corresponding author on request. The crystallographic data have been deposited at the Cambridge Crystallographic Data Centre with reference number CCDC 2150690-2150694. This data can be obtained free of charge from The Cambridge Crystallographic Data Centre at http://www.ccdc.cam.ac.uk/data_request/cif.
