# Supplementary material for: Ultrafiltration separation of Am(VI)-polyoxometalate from lanthanides
Source: Nature. 2023 Apr 19;616(7957):482–7. doi: 10.1038/s41586-023-05840-z (PMC10115636; doi:10.1038/s41586-023-05840-z)
Supplement: Supplementary file 1 — Supplementary Sections 1–22, including Figs. 1–40, Tables 1–12 and References. [file 41586_2023_5840_MOESM1_ESM.docx]

Supplementary Information

**Ultrafiltration separation of Am(VI)-polyoxometalate from lanthanides**

Hailong Zhang^1^†, Ao Li^1^†, Kai Li^1^†, Zhipeng Wang^2^, Xiaocheng Xu^3^, Yaxing Wang^1^*, Matthew V. Sheridan^1^, Han-Shi Hu^3^, Chao Xu^2^*, Evgeny V. Alekseev^4^, Zhenyi Zhang^5^, Pu Yan^6^, Kecheng Cao^6^, Zhifang Chai^1^, Thomas E. Albrecht-Schönzart^7^*, Shuao Wang^1^*

*^1^State Key Laboratory of Radiation Medicine and Protection, School for Radiological and interdisciplinary Sciences (RAD-X) and Collaborative Innovation Center of Radiation Medicine of Jiangsu Higher Education Institutions, Soochow University, Suzhou 215123, China*

*^2^Institute of Nuclear and New Energy Technology, Tsinghua University, Beijing 100084, China*

*^3^Department of Chemistry and Laboratory of Organic Optoelectronics & Molecular Engineering of the Ministry of Education, Tsinghua University, Beijing 100084, China*

*^4^IEK-9, Forschungszentrum Jülich, Wilhelm-Johnen-Str. 52428 Jülich, Germany*

*^5^Bruker (Beijing) Scientific Technology Co., Ltd, Shanghai 200233, China*

*^6^Shanghai Key Laboratory of High-resolution Electron Microscopy, ShanghaiTech University, Shanghai, 201210, China*

*^7^Department of Chemistry and Nuclear Science and Engineering Center, Colorado School of Mines, Golden, Colorado 80401, United States*

*†These authors contributed equally to this work.*

Email: yxwang@suda.edu.cn (Y. W.); xuchao@tsinghua.edu.cn (C. X.); tschoenzart@mines.edu (T. E. A.-S.); shuaowang@suda.edu.cn (S. W.).

**This Supplementary Information includes:**

**Supplementary Figures 1 to 40**

**Supplementary Tables 1 to 12**

**Table of Contents**

1. Structural evolution of POM and solution chemistry of POM. **Supplementary Fig. 1-S3**

2. Species distribution curves as a function of the POM/Am ratio during titration. **Supplementary Fig. 4**

3. Solution chemistry investigation of Ln(III) with {Se_6_W_45_}. **Supplementary Figs. 5 and 6**

4. Electrochemistry investigation of the POM and the Am(VI)-POM. **Supplementary Fig. 7-11**

5. Microscopic photos of single crystals for POM and An(VI)-POM. **Supplementary Fig. 12**

6. Electron microscopy studies. **Supplementary Fig. 13-15**

7. Computation results. **Supplementary Fig. 16-18**

8. Condition optimization of the separation experiment. **Supplementary Fig. 19-21**

9. Energy spectra data for the feed solution, the retentate solution, and the permeate solution. **Supplementary Fig. 22**

10. Preparation of hexavalent transuranium stock solutions. **Supplementary Fig. 23-25**

11. TGA, EDS measurement, and UV‒Vis spectroscopy data. **Supplementary Fig. 26-29**

12. Synthetic procedures and unit cells of POM and An(VI)-POM crystals. **Supplementary Fig. 30**

13. The refinement procedure and results for positional disorder of POM and An(VI)-POM. **Supplementary Fig. 31-40**

14. Comparisons on the reported values on the binding constant (logβ) between actinyl(VI) ions and inorganic ligands in aqueous solution. **Supplementary Table 1**

15. The change in luminescence lifetime (*τ*) and inner-sphere coordinated number of Eu(III) during the titration. **Supplementary Table 2**

16. Redox peak potentials for the tungsten or Americium waves determined by cyclic voltammetry. **Supplementary Table 3**

17. Selected bond lengths of An-O in An(VI)-POM. **Supplementary Table 4**

18. EDA-NOCV energies of An(VI)-POMs and Ln(H_2_O)_8_ at the PBE/TZ2P/TZP/DZP level. **Supplementary Table 5**

19. BVS calculation results of POM and An-POM. **Supplementary Table 6-10**

20. Elemental analysis for POM and U(VI)-POM. **Supplementary Table 11**

21. Crystallographic data and structure refinement parameters for POM and An(VI)-POM. **Supplementary Table 12**

22. **References**

# Supplementary Figures

# 1. Structural evolution of POM and solution chemistry of POM


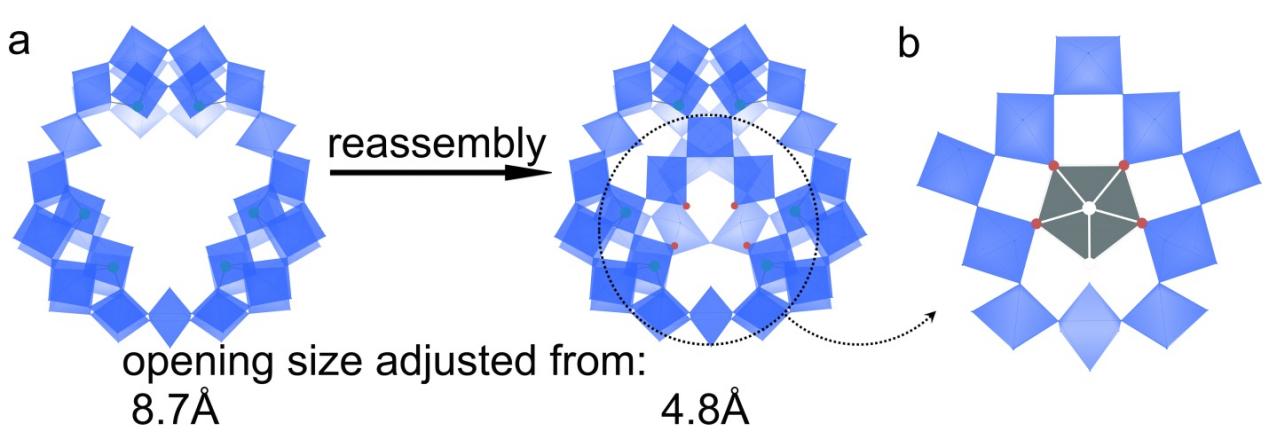


**Fig. 1 a.** The cluster {Se_6_W_45_} was obtained by reassembly of {Se_6_W_39_} in acid solution, with the aperture changed from 8.7Å to 4.8Å, forming a new vacancy site. **b**. The vacancy site with four planar oxygen ligands for binding actinyl ions. In the cap of the POM, three WO_6_^6-^group are resolved with severe positional disorder issue. The three configurations of {W_3_O_16_} are illustrated in Figs S20 and S21. A single configuration contains a vacancy site with an aperture size of 4.8 Å, leaving a preorganized coplanar oxo-donor structure for binding actinyl ions.





**Fig. 2** Volume size distribution on the cluster {Se_6_W_45_} (1 mg/mL) in 0.1 M nitric acid solution.





**Fig. 3** Raman spectra of {Se_6_W_45_} in the solid state and in 0.1 M nitric acid solution (50 mg/mL).

# 2. Species distribution curves as a function of the POM/Am ratio during titration


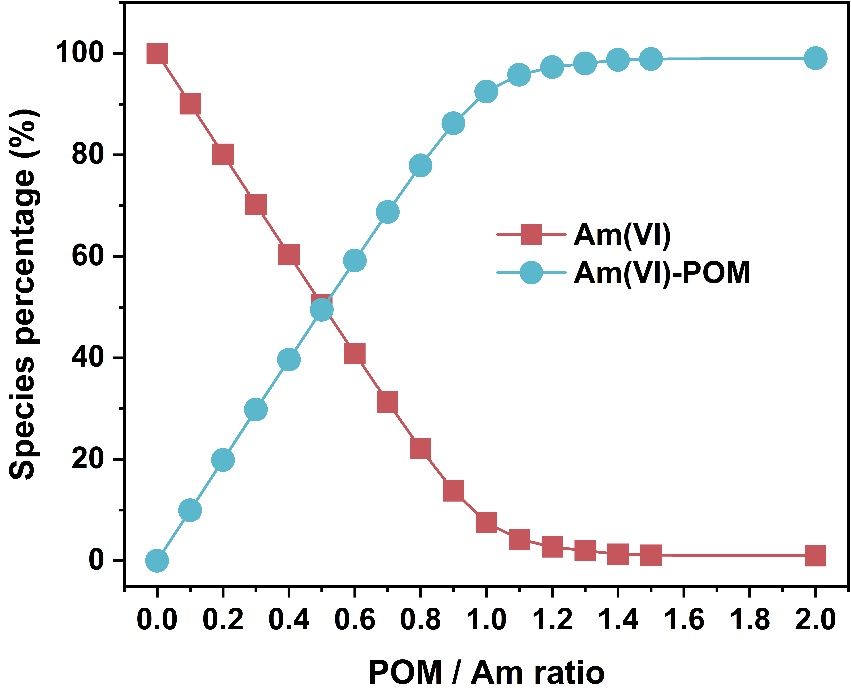


**Fig. 4** The species distribution curves as a function of the POM/Am ratio during titration.

# 3. Solution chemistry investigation of Ln(III) with {Se_6_W_45_}


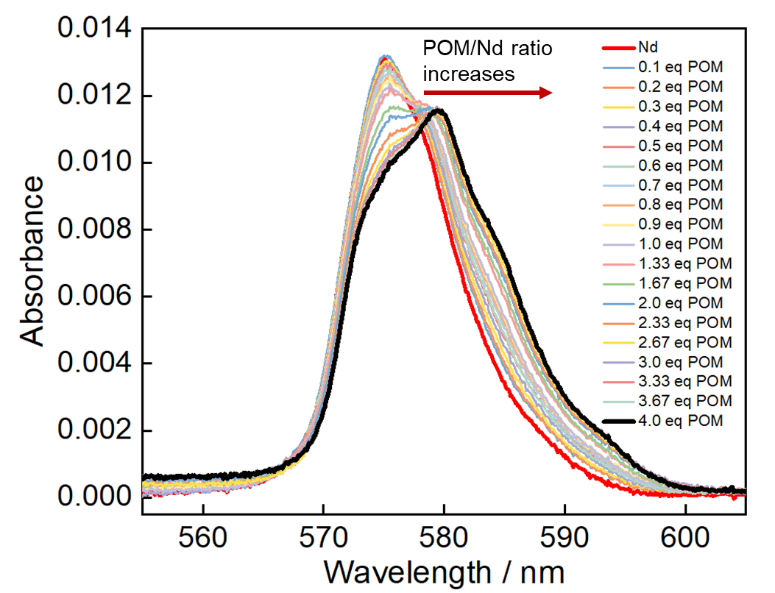


**Fig. 5** Spectrophotometric titration of Nd(III) with POM. Initial solution: *V*_0_ = 1.5 mL, [Nd]_0_ = 2 mM, [HNO_3_] = 0.1 M, 10 mm cuvette. Titrant: 10 mM POM in 0.1 M HNO_3_. With the addition of POM into the Nd(III) solution, the characteristic 4f−4f transition (^4^I_9/2_ → ^4^G_5/2_) absorption band of Nd(III) red-shifts gradually toward longer wavelengths, indicating that there are interactions between Nd(III) and POM. The spectra were further analyzed by the Hypspec program to obtain more quantitative results. The analysis suggests that only one Nd/POM complex with a stoichiometry of 1:2 formed during the titration. The obtained stability constants (log*β*_12_ = 5.3 ± 0.1) for the 1:2 complex suggest that the interaction between Nd(III) and POM is quite weak. As contrasted with the least stable 1:2 Eu(III)^/^GeW_11_O_39_^8-^ complex in the literature (*Inorganica Chimica Acta*, **2003**, *346*, 215-222), whose stability constant (log*β*_12_ = 11.1 ± 1.3) is more than 5 orders larger than that (log*β*_12_ = 5.3 ± 0.1) of the 1:2 Nd/POM complex in this work.


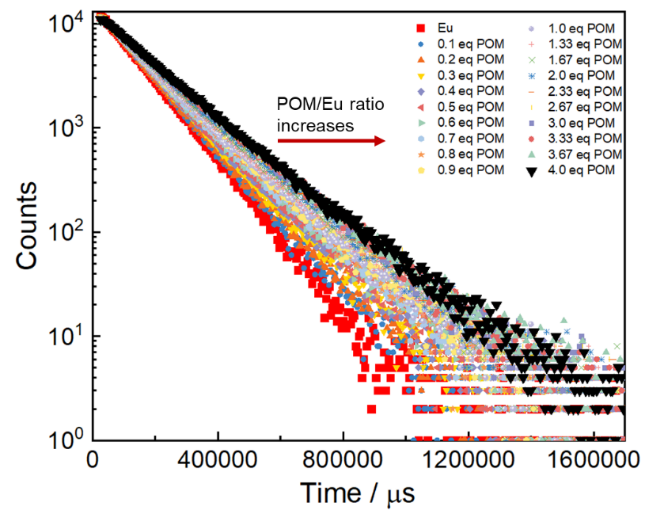


**Fig. 6** Luminescence lifetime titration of Eu(III) with POM. Initial solution: *V*_0_ = 1.5 mL, [Eu]_0_ = 2 mM, [HNO_3_] = 0.1 M, 10 mm cuvette. Titrant: 10 mM POM in 0.1 M HNO_3_. Upon addition of POM into the Eu(III) solution, the decay of Eu(III) luminescence becomes slower. All decays exhibit a monoexponential feature, suggesting that there are fast exchanges between different Eu(III) species in the solution. This also indicates the weak coordinating nature between Eu(III) and POM. The data were fitted to obtain the lifetime values and the number of water molecules (*N*_H2O_) in the inner coordination sphere of Eu(III) through the established relationship *N*_H2O_ = 1.05/*τ* - 0.44. As shown in Table S4, the Eu(III) ion is still mostly hydrated, and fewer than 3 water molecules are replaced even in the presence of 4 equivalents of POM in the solution. This indicates that that lanthanide ions complex with POM mainly through relatively weak electrostatic interactions between the hydrated Ln ions and the negatively charged POM anions but not through inner-sphere coordination by forming a direct bond between Ln and atoms in the lacunary hole in the POM.

#

# 4. Electrochemistry investigation of the POM and the Am(VI)-POM


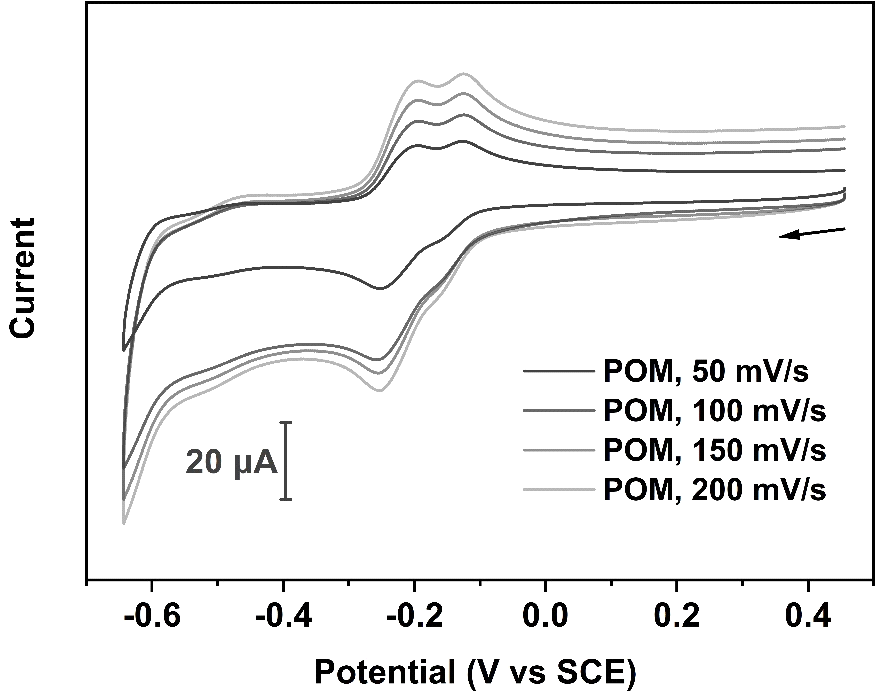


**Fig. 7** Cyclic voltammograms of 0.5 mM POM in 0.5 M Na_2_SO_4_/ 0.05 M H_2_SO_4_ buffer solution with pH=1.0. The scan rate varies from 50 to 200 mV/s. The working electrode was glassy carbon (3 mm) and the reference was a SCE.


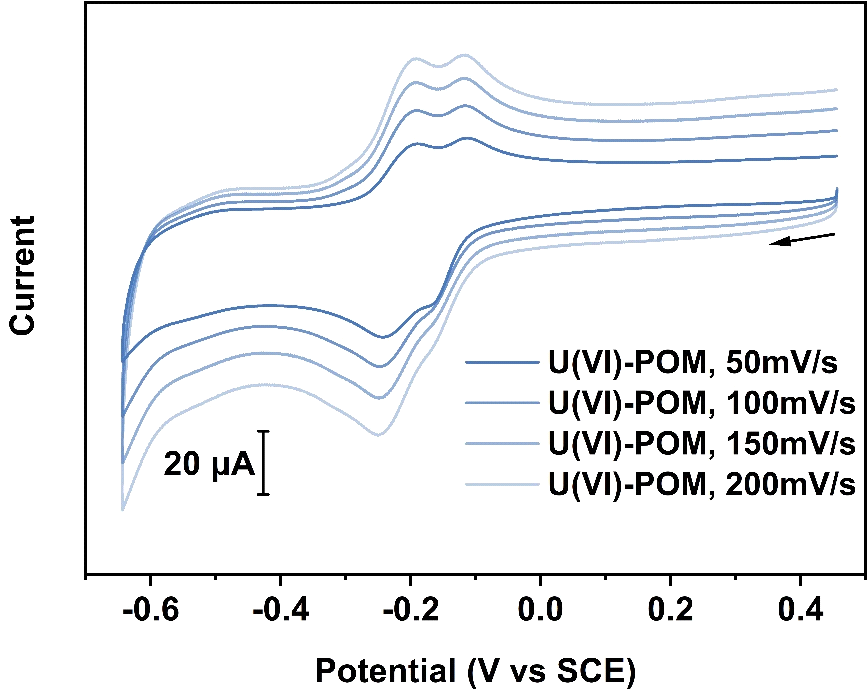


**Fig. 8** Cyclic voltammograms of 0.5 mM U(VI)-POM in 0.5 M Na_2_SO_4_/ 0.05 M H_2_SO_4_ buffer solution with pH=1.0. The scan rate varies from 50 to 200 mV/s. The working electrode was glassy carbon (3 mm) and the reference was a SCE.


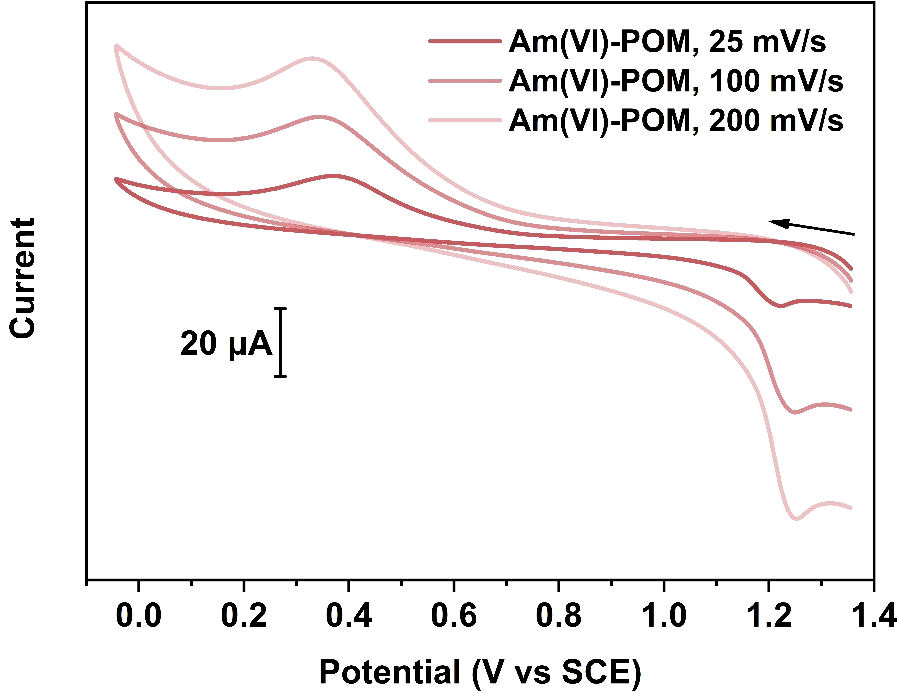


**Fig. 9** Cyclic voltammograms of 0.5 mM Am(VI)-POM in 0.5 M Na_2_SO_4_/ 0.05 M H_2_SO_4_ buffer solution with pH=1.0. The scan rate varies from 25 to 200 mV/s. The working electrode was glassy carbon (3 mm) and the reference was a SCE.


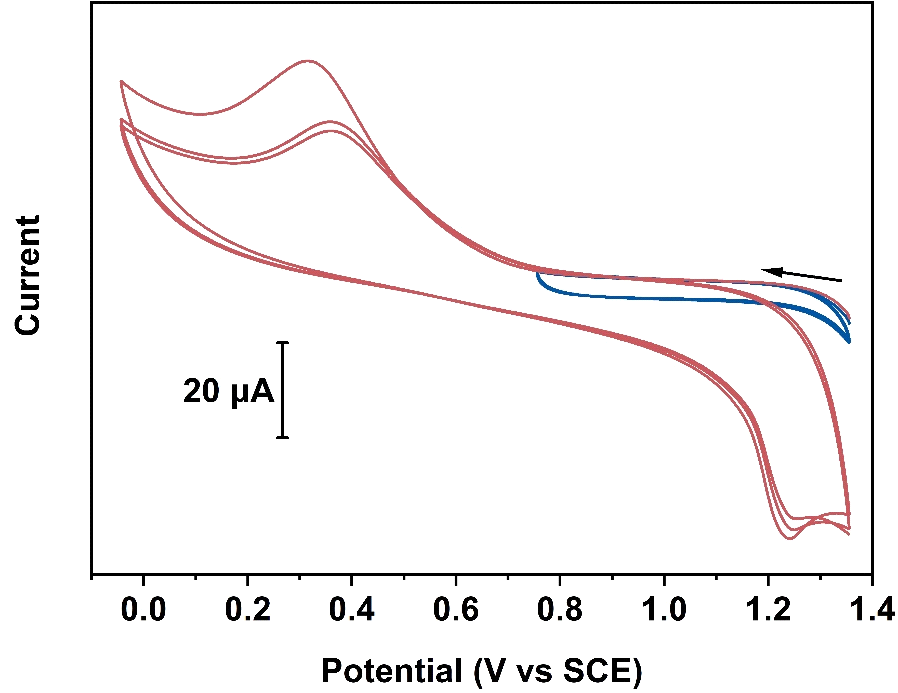


**Fig. 10** Cyclic voltammograms of 0.5 mM Am(VI)-POM in 0.5 M Na_2_SO_4_/ 0.05 M H_2_SO_4_ buffer; switching before (blue) and after (red) the Am(VI)-POM is reduced; The scan rate was 100 mV/s. The working electrode was glassy carbon (3 mm) and the reference was a SCE.


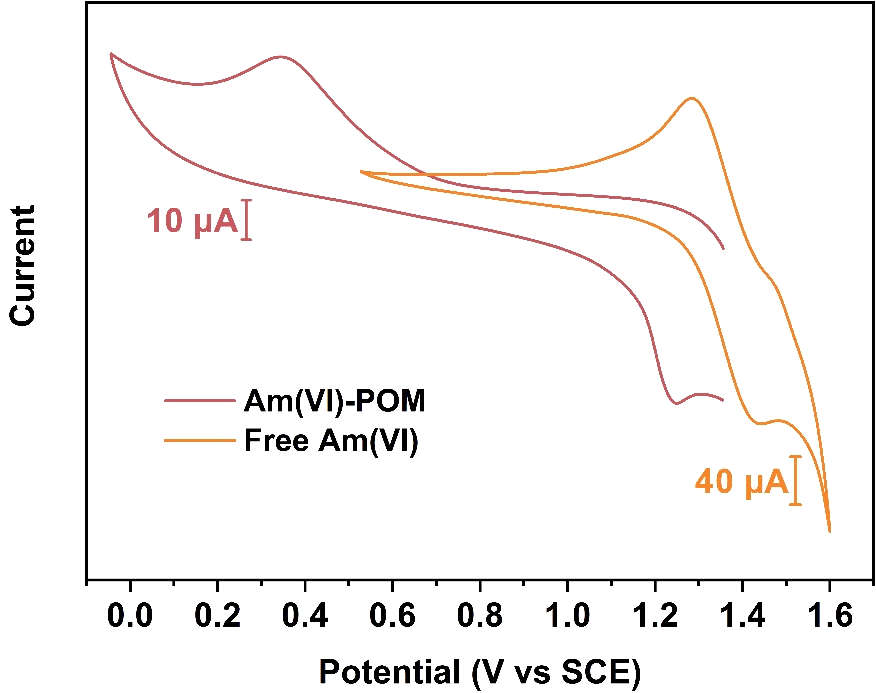


**Fig. 11** Cyclic voltammograms of 0.5 mM Am(VI)-POM in 0.5 M Na_2_SO_4_/ 0.05 M H_2_SO_4_ buffer solution with pH=1.0, and that of 0.01 M free Am(VI) in 0.1 M HNO_3_. The scan rate was 100 mV/s. The working electrode was glassy carbon (3 mm) and the reference was a SCE.

# 5. Microscopic photos of single crystals for POM and An(VI)-POM

**
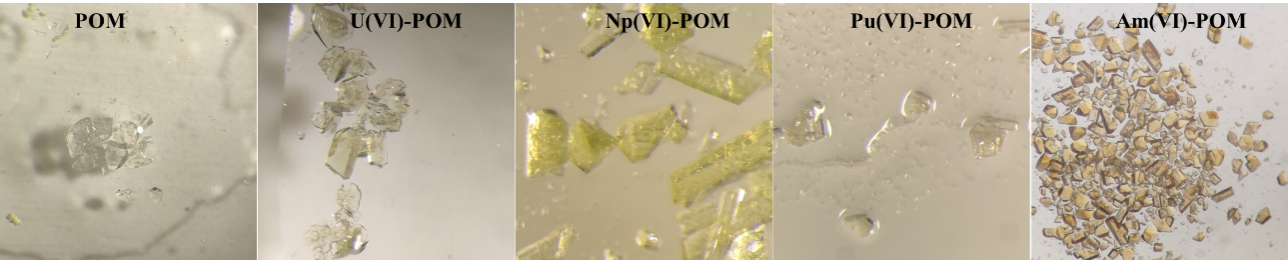
**

**Fig. 12** The microscopic photos of single crystals for POM and An(VI)-POM.

# **6.** Electron microscopy studies


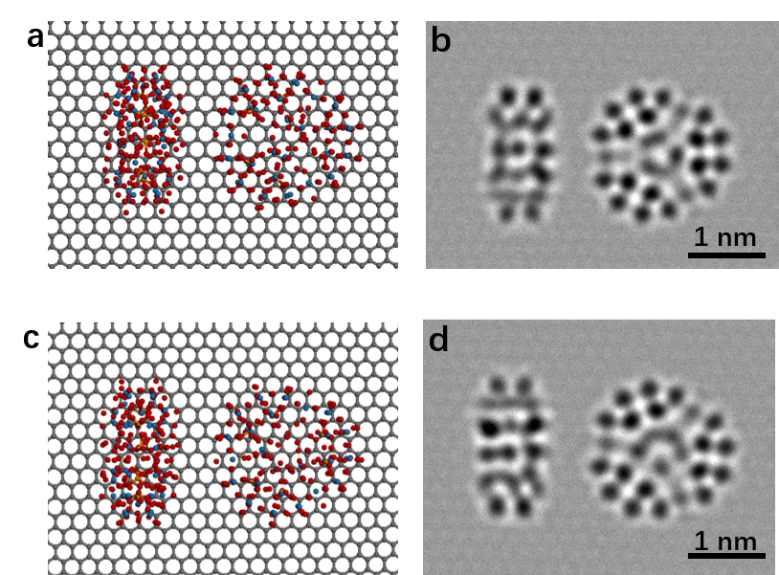


**Fig. 13** TEM image simulation of POM and U(VI)-POM by QSTEM. **a,** model of a standing and a lying POM on single layer graphene. **b,** simulated TEM image based on the model in **a** by QSTEM using the same TEM conditions during experiments. **c,** model of a standing and a lying U(VI)-POM on single layer graphene. **d,** simulated TEM image based on the model in **c** by QSTEM using the same TEM conditions during experiments. According to the orientations of POM and U(VI)-POM, they showed different morphologies.


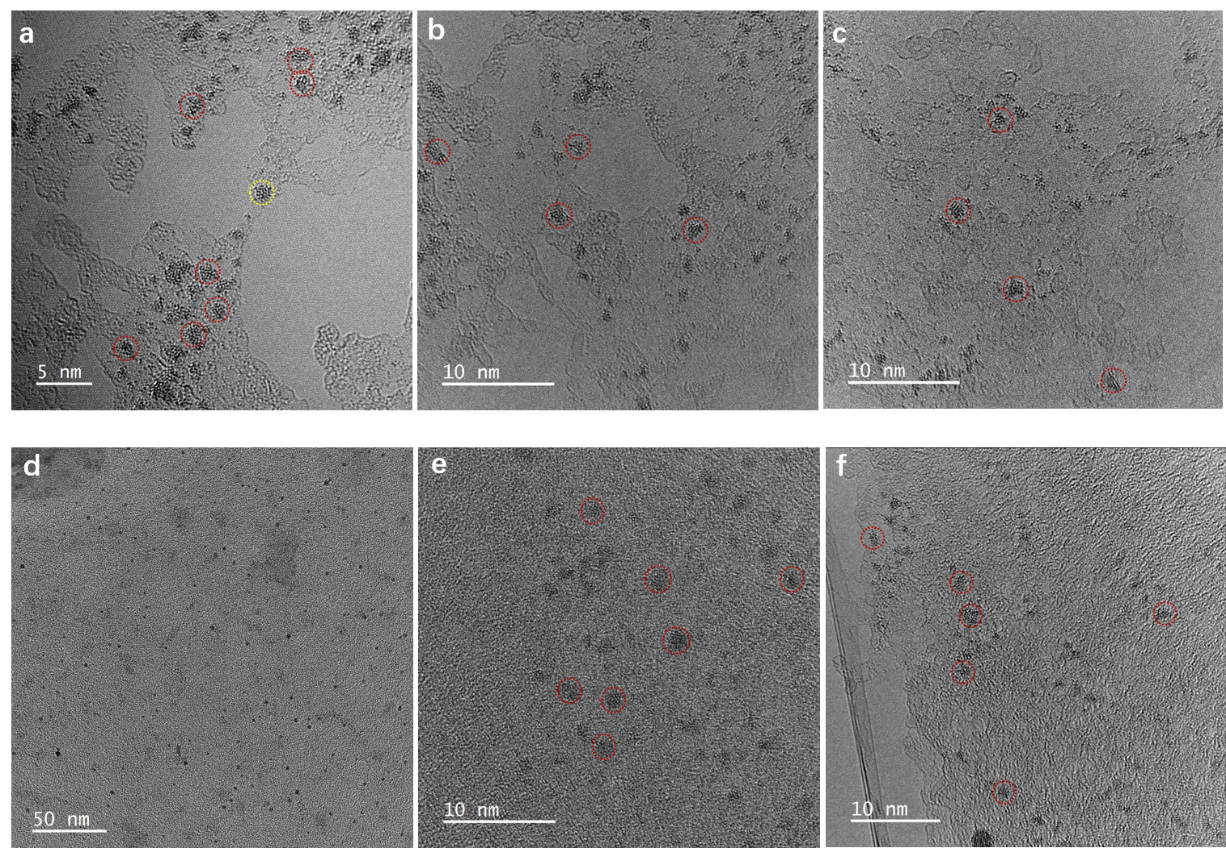


**Fig. 14** Raw ACTEM images of POM and U(VI)-POM on graphene. **a-c,** ACTEM images of POM on graphene. **d-f,** typical ACTEM images of U(VI)-POM on graphene. The POM and U(VI)-POM tend to adhere the amorphous carbon on graphene owing to the relatively abundant polarized chemical bonds comparing with graphene. Some typical POM and U(VI)-POM are highlighted by circles according to their size and structure. The POM highlighted by yellow circle is further studied in Fig. 3 in the main text. The imaged POM and U(VI)-POM show different morphologies owing to their different orientations on the graphene subtract and electron beam damage during TEM investigation. The dynamics POM and U(VI)-POM under electron beam irradiation are observed, including their dissociation, aggregation and reaction with substrate.


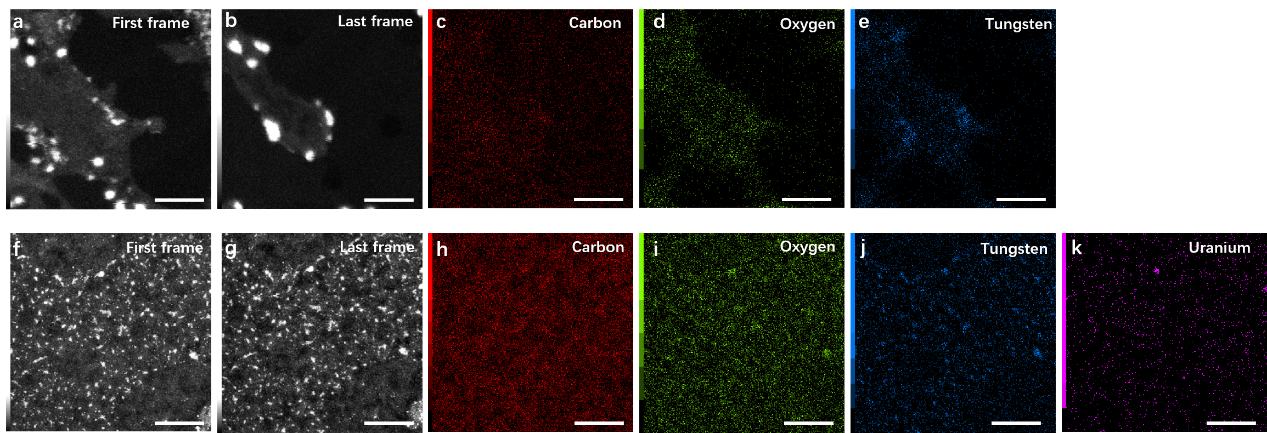


**Fig. 15** EDS mapping of POM and U(VI)-POM. **a - e,** EDS mapping of POM, showing the distribution of C, O and W. **f - k,** EDS mapping of U(VI)-POM, showing the distribution of C, O, W, and U. The POM and U(VI)-POM are unstable under electron beam irradiation during EDS investigation.

# **7.** Computation results





**Fig. 16** The calculated total orbital interactions for actinyl-POM and lanthanide-POM.


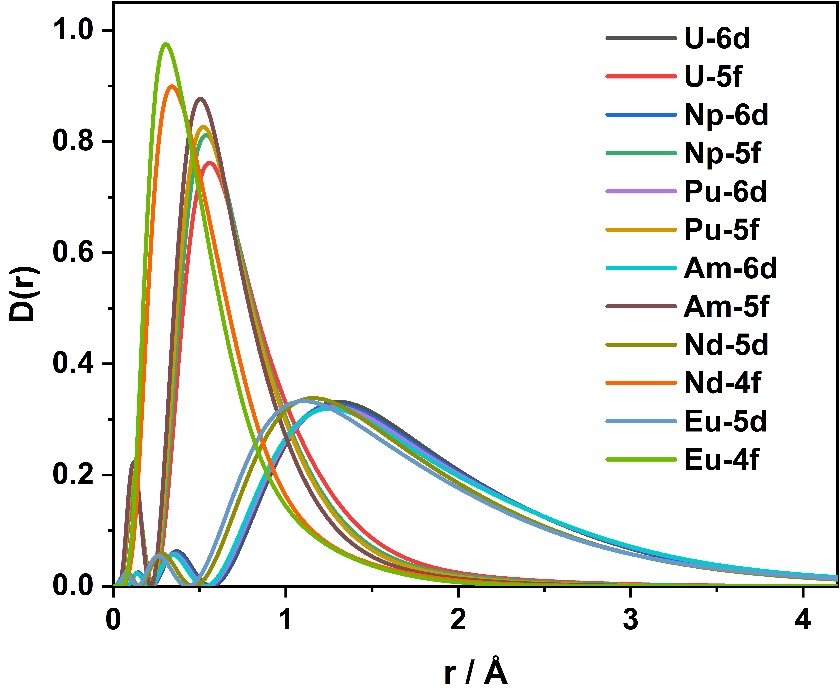


**Fig. 17** The radial densities of the 4f atomic orbitals in the lanthanide elements and 5f atomic orbitals of the actinide elements.


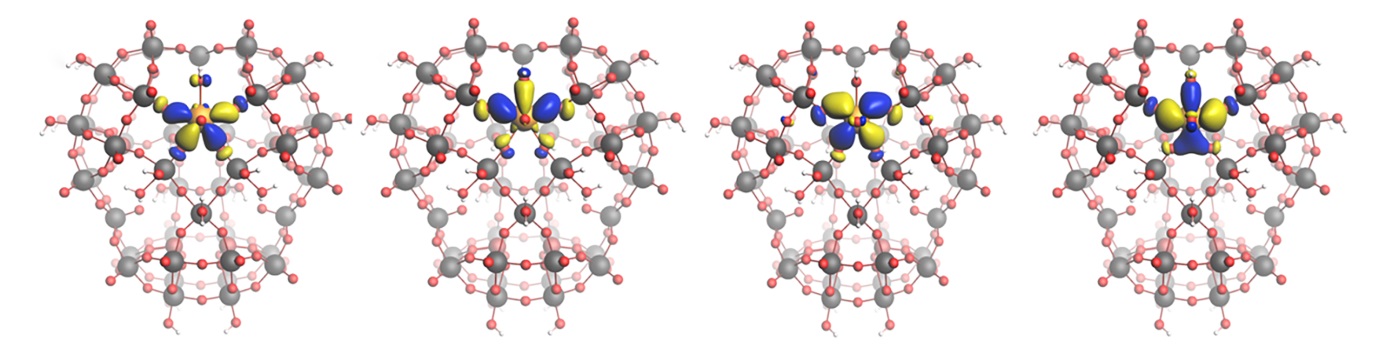


**Fig. 18** The energy decomposition analysis of Am(VI)-POM.

# **8. Condition Optimization of the separation experiment**





**Fig. 19** The influence of acidity on the separation factor (SF) and rejection coefficient (%) for U(VI)/Eu(III) binary separation. The reaction conditions: initial U(VI) concentration is 2.0 ×10^-5^ mol/L; initial Eu(III) concentration is 2.0 ×10^-5^ mol/L; POM concentration is 4.0 ×10^-5^ mol/L; NH_4_NO_3_ concentration is 0.15 mol/L; reaction time is 5 min.





**Fig. 20** The influence of NH_4_^+^ ions on the separation factor (SF) and rejection coefficient (%) for U(VI)/Eu(III) binary separation. The reaction conditions: initial U(VI) concentration is 2.0 ×10^-5^ mol/L; initial Eu(III) concentration is 2.0 ×10^-5^ mol/L; POM concentration is 4.0 ×10^-5^ mol/L; HNO_3_ concentration is 0.1 mol/L; reaction time is 5 min.





**Fig. 21** The influence of reaction time on the separation factor (SF) and rejection coefficient (%) for U(VI)/Eu(III) binary separation. The reaction conditions: initial U(VI) concentration is 2.0 ×10^-5^ mol/L; initial Eu(III) concentration is 2.0 ×10^-5^ mol/L; POM concentration is 4.0 ×10^-5^ mol/L; HNO_3_ concentration is 0.1 mol/L; NH_4_NO_3_ concentration is 0.15 mol/L.

# **9.** Energy spectra data for the feed solution, the retentate solution, and the permeate solution


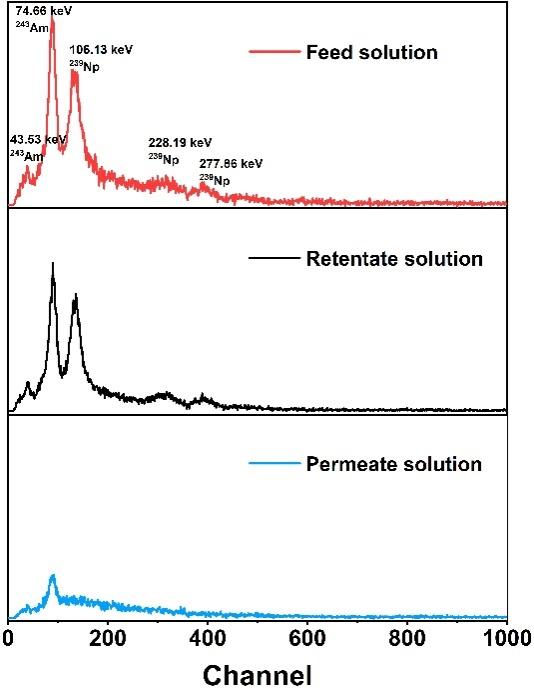


**Fig. 22** Energy spectra of a feed solution containing ^243^Am (top) and trace amount of the decay daughter ^239^Np, the retentate solution after ultrafiltration separation (middle), and the permeate solution after ultrafiltration separation (bottom). The peak intensity in the γ energy spectrum is only related to the probability of emission (since the activities of ^243^Am and ^239^Np are considered to be the same according to the theory of secular radioactive equilibrium). The probability of emission of ^243^Am at 74.644 keV is 68%, and the probability of emission of ^239^Np at 106.125 keV is 27.2%. It can be roughly concluded that the ratio is approximately 68/27.2=2.5, consistent with the peak-area ratio in Fig. S22. Besides, according to the theory of secular radioactive equilibrium, the ratio of the concentration of ^243^Am (half-life of 7380 y) and ^239^Np (half-life of 2.356 d) in solution is inversely proportional to the ratio of its decay constant (λ = ln2/T, T is the half-life). The molar ratio of ^243^Am:^239^Np was calculated to be ~1143336 in the ^243^Am mother liquor, and ^239^Np can be therefore considered to be in trace amount.

# 10. Preparation of hexavalent transuranium stock solutions


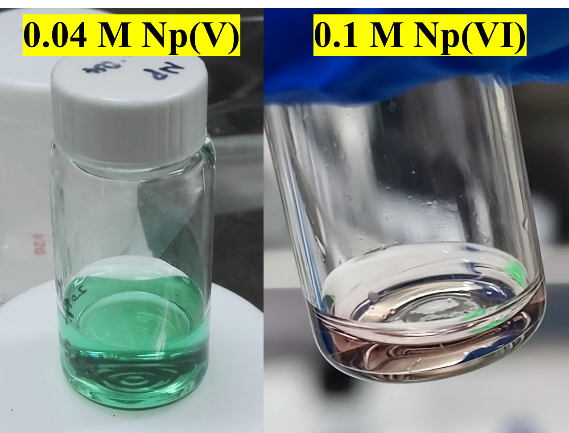


**Fig. 23.** The stock solutions of Np(V) perchlorate and Np(VI) nitrate.

**Fig. 24.** Preparation of 0.1 M Pu(VI) nitrate solution.


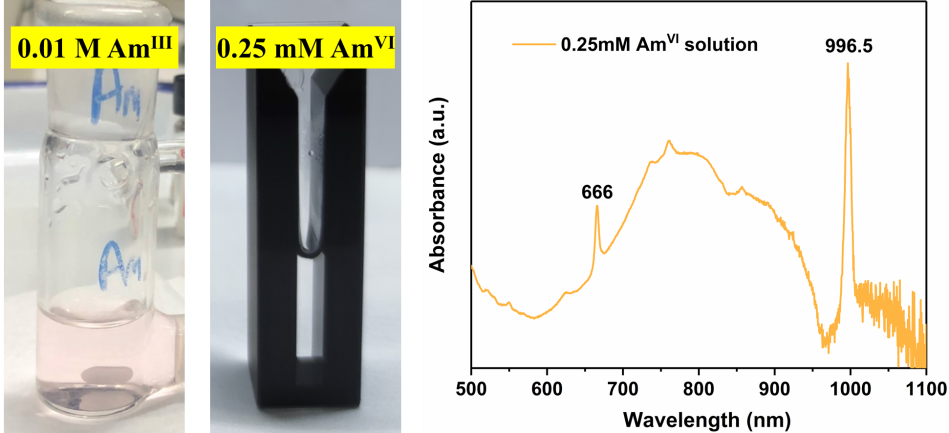


**Fig. 25.** Preparation of 0.25 mM Am(VI) nitrate solution.

# 11. TGA, EDS measurement, and UV‒Vis spectroscopy data


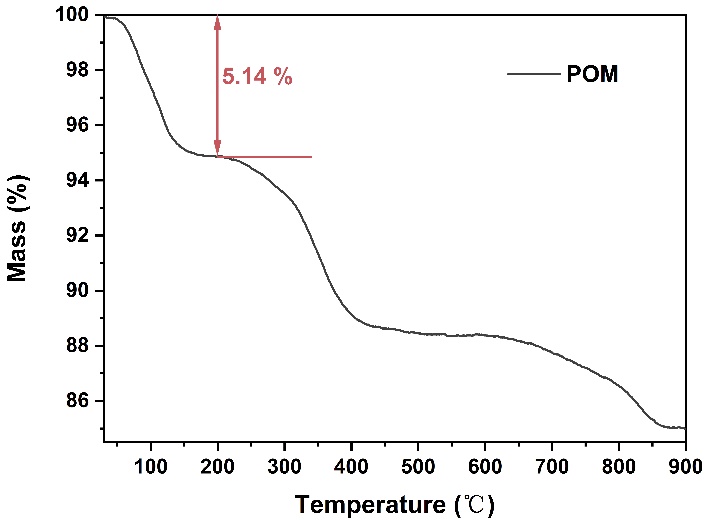


**Fig. 26** TGA measurement result of POM showing the amount of water loss from 30 to 200 °C.


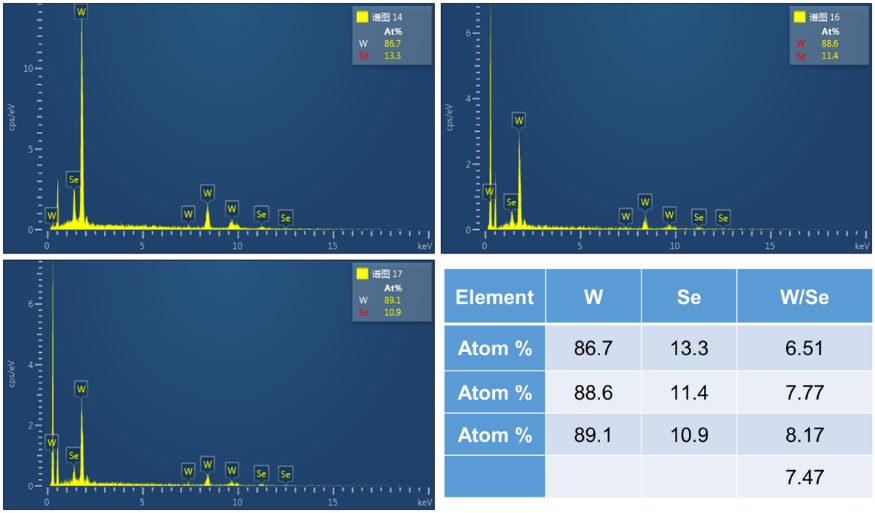


**Fig. 27** The W/Se molar ratio in POM crystal determined by energy dispersive spectrometry (EDS).


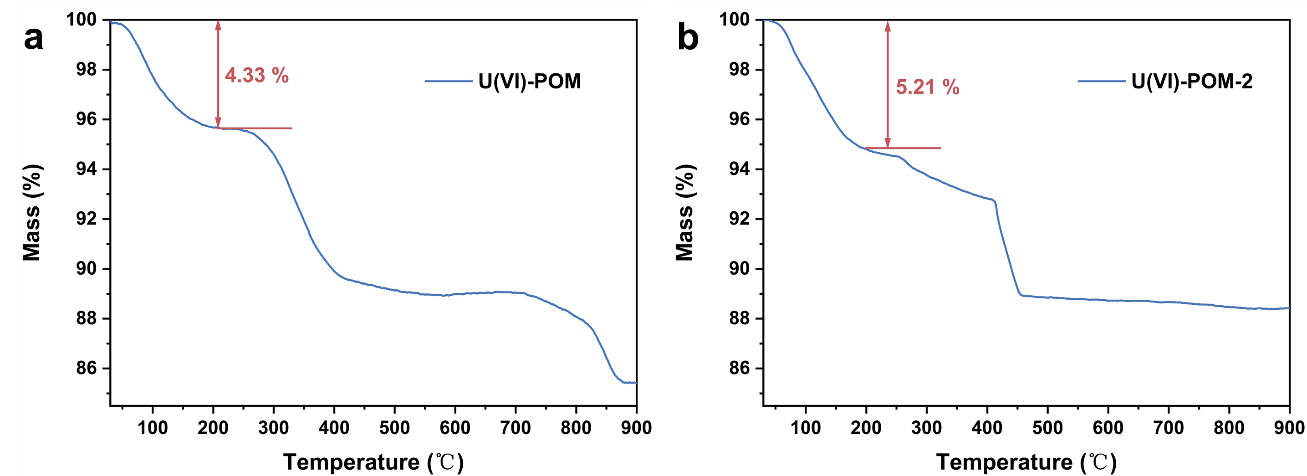


**Fig. 28** TGA measurement result of U(VI)-POM showing the amount of water loss from 30 to 200 °C.


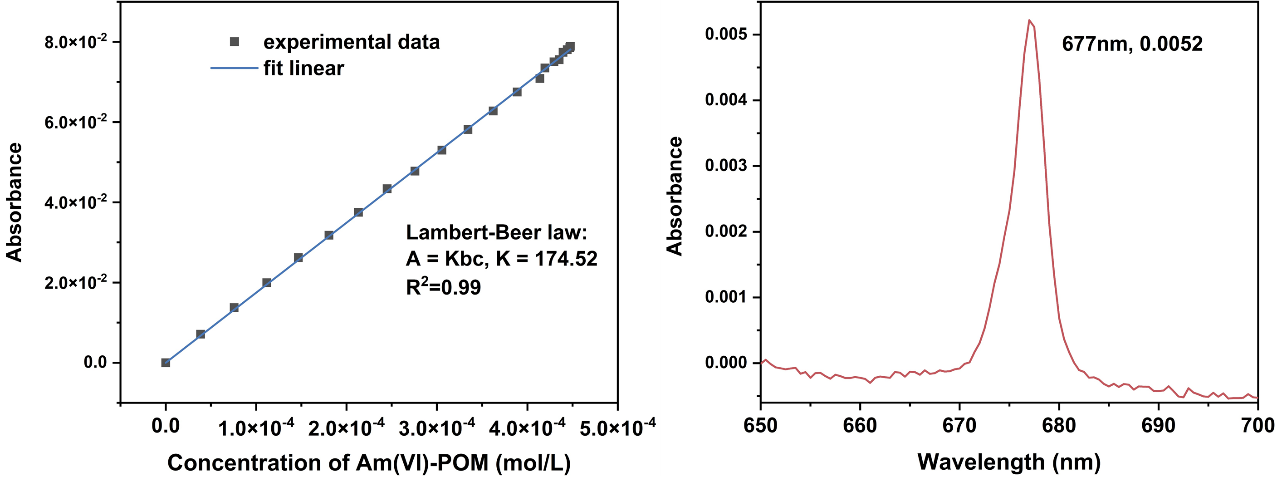


**Fig. 29** Lambert‒Beer law: A = K*b*c, A is absorbance, K is molar extinction coefficient, unit is M^-1^·cm^-1^; b is optical path length, b = 1, unit is cm; c is the concentration, unit is M. Based on the fitting result, the molar absorptivity of Am(VI)-POM species at 677 nm is 174.52 M^-1^·cm^-1^. The concentration of the Am(VI)-POM complex was calculated to be 2.97×10^-5^ M. In addition, the Am concentration was determined by liquid scintillation counting (LSC) (0.4 mg Am(VI)-POM crystals was dissolved and then taken at a certain volume for the LSC test). The calculated concentration of Am in the solution based on LSC is 3.01×10^-5^ M, (c = cpm/t/a/M/V, cpm is liquid scintillation counting, the measured counting is 32465, t is count time, a is specific-activity, a(^243^Am) = 7.39 × 10^9^ Bq/g, V is the sample volume). Therefore, we can conclude that the ratio of Am: POM in the Am(VI)-POM complex is very close to 1:1.

# **12.** Synthetic procedures and unit cells of POM and An(VI)-POM crystals


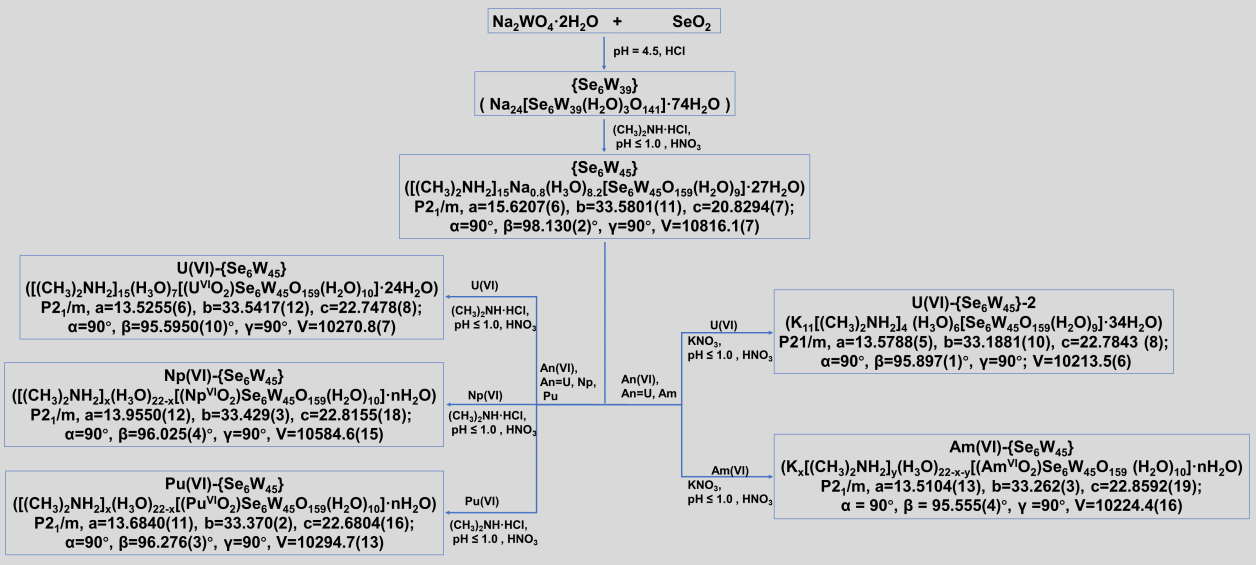


**Fig. 30** Summary of the synthetic procedures for POM and An(VI)-POM crystals.

# 13. The refinement procedure and results for positional disorder of POM and An(VI)-POM


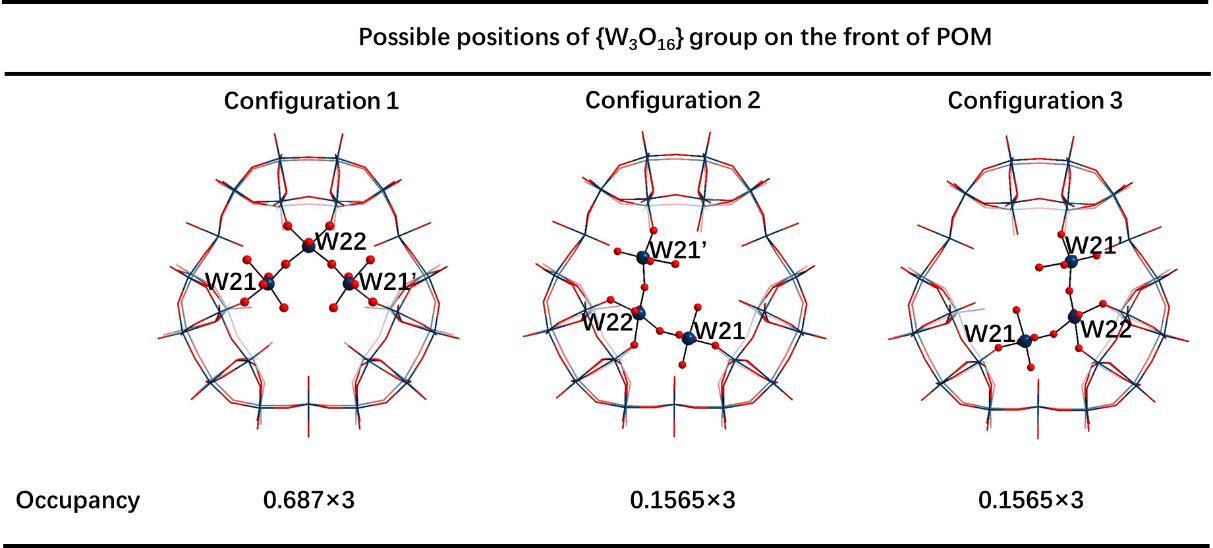


**Fig. 31** The positional disorder of {W_3_O_16_} group around the hole of POM.


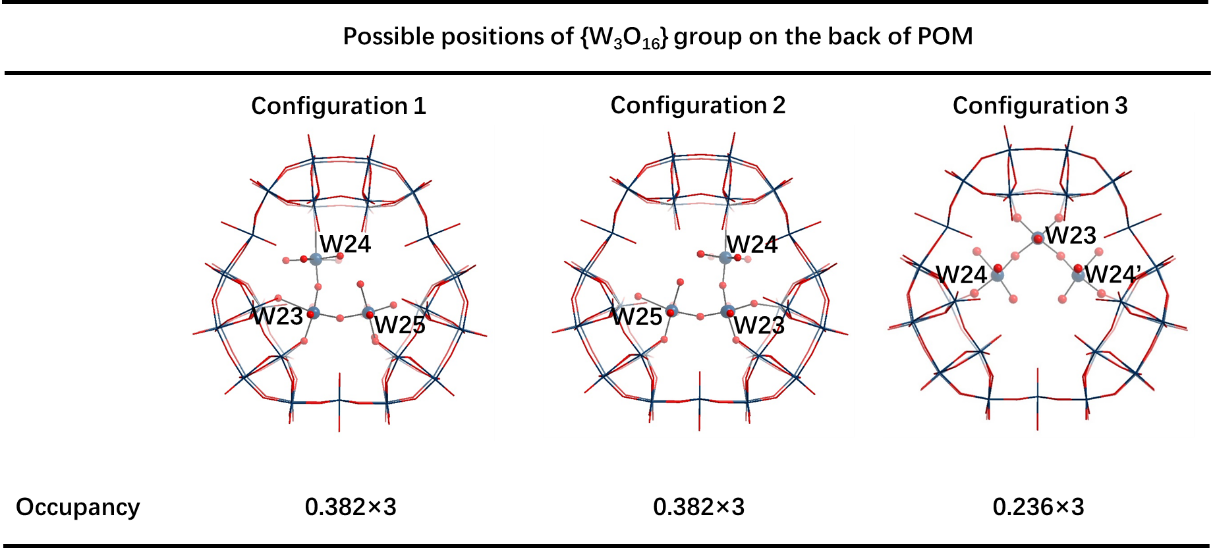


**Fig. 32** The positional disorder of {W_3_O_16_} group on the opposite face of POM. On the opposite face of the POM, there are also three equivalent sites that is positional disorder. Structure refinement found the SOF of W atoms are 1.5 in the asymmetric unit and 3 in a POM unit. Therefore, it is also a disorder of {W_3_O_16_} group (consisting of W23, W24 and W25).


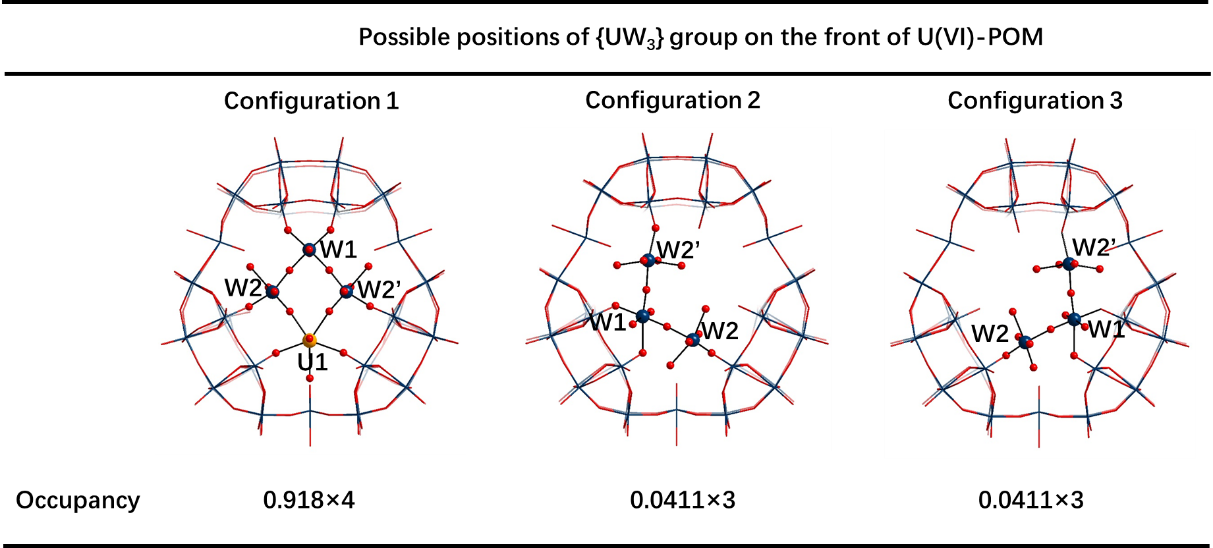


**Fig. 33** The positional disorder of {UW_3_} group over three positions on the front of U(VI)-POM.


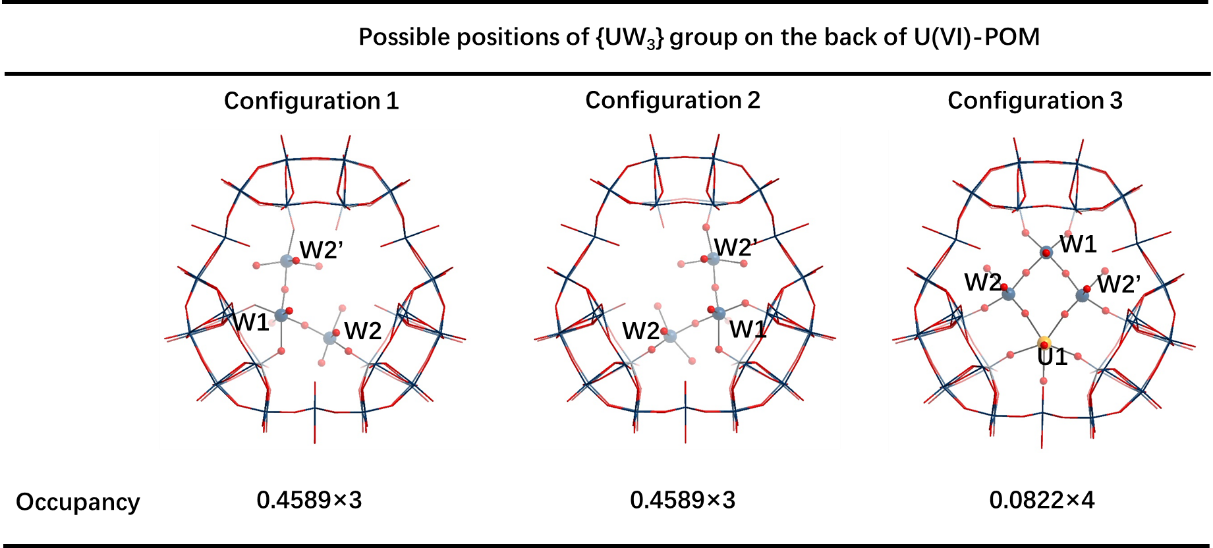


**Fig. 34** The positional disorder of {UW_3_} group on the opposite face of U(VI)-POM.


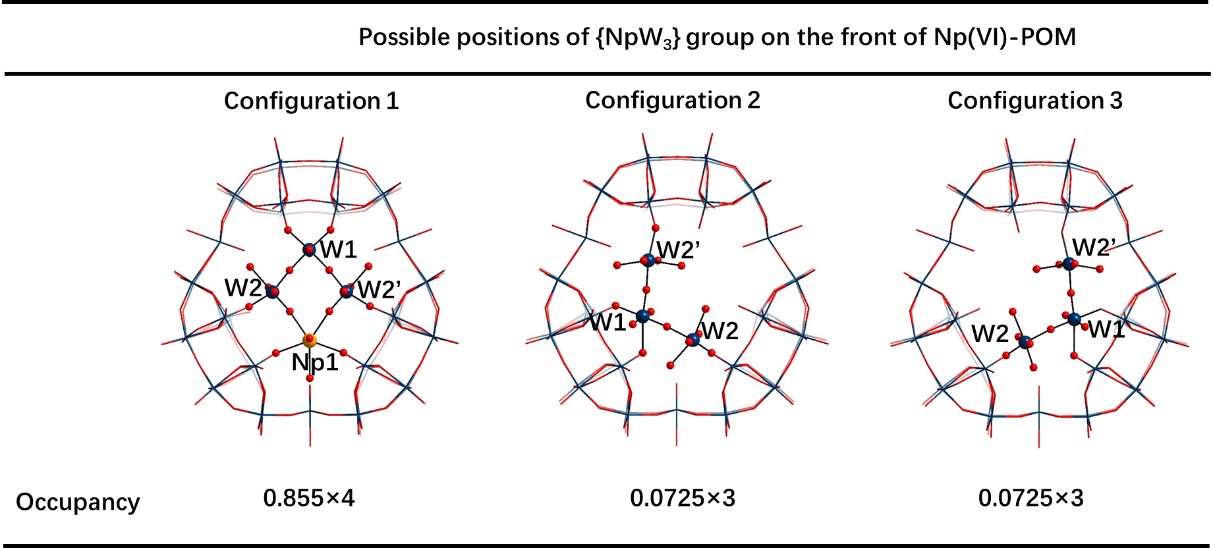


**Fig. 35** The positional disorder of {NpW_3_} group over three positions on the front of Np(VI)-POM.


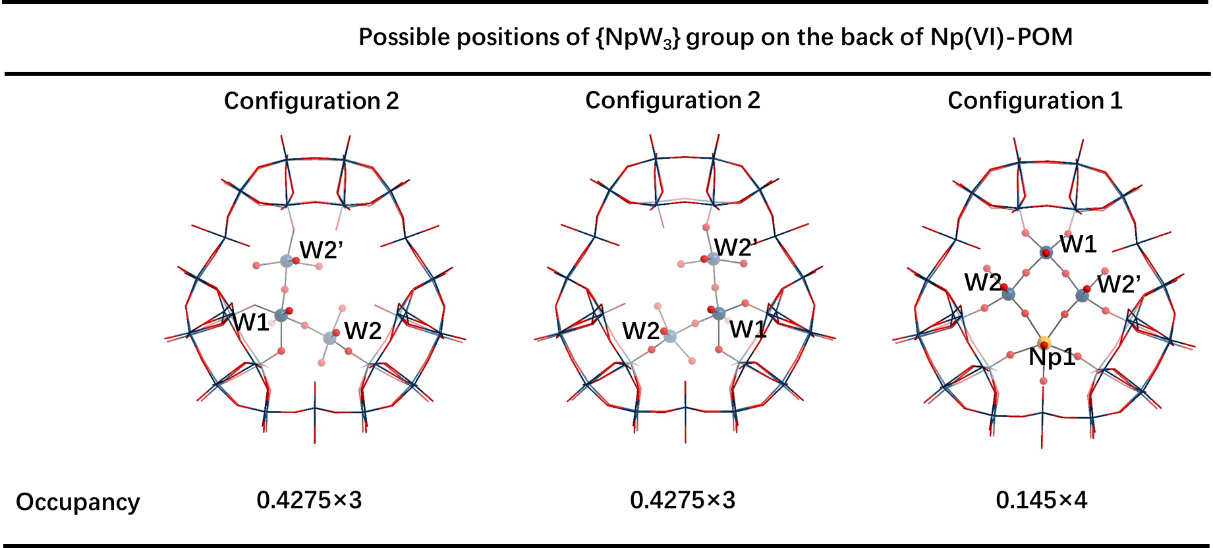


**Fig. 36** The positional disorder of {NpW_3_} group is disordered over three positions on the opposite face of Np(VI)-POM.


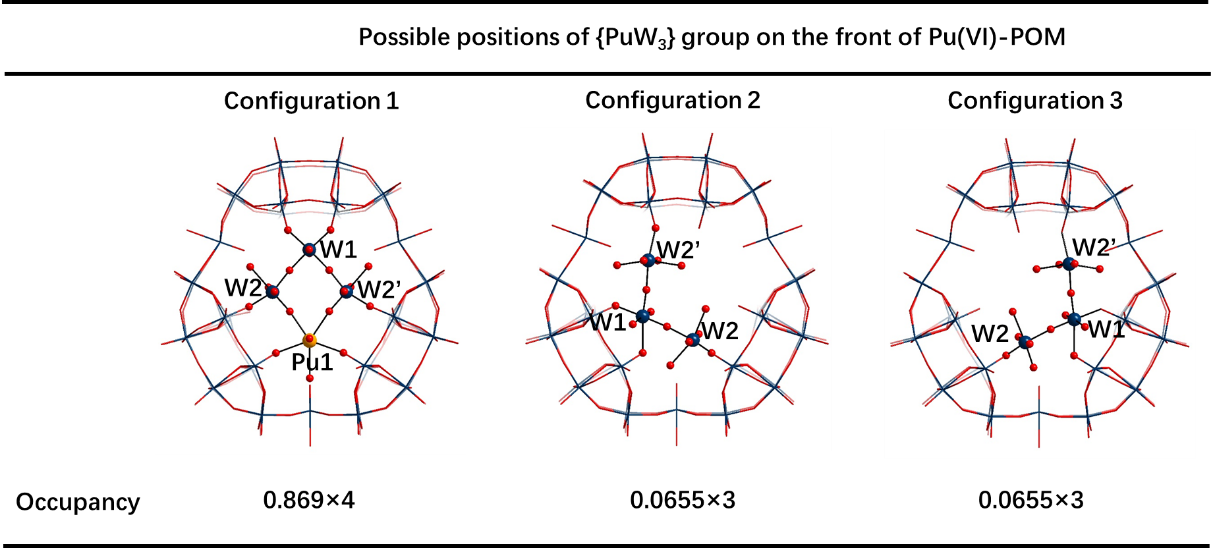


**Fig. 37** The positional disorder of {PuW_3_} group over three positions on the front of Pu(VI)-POM.


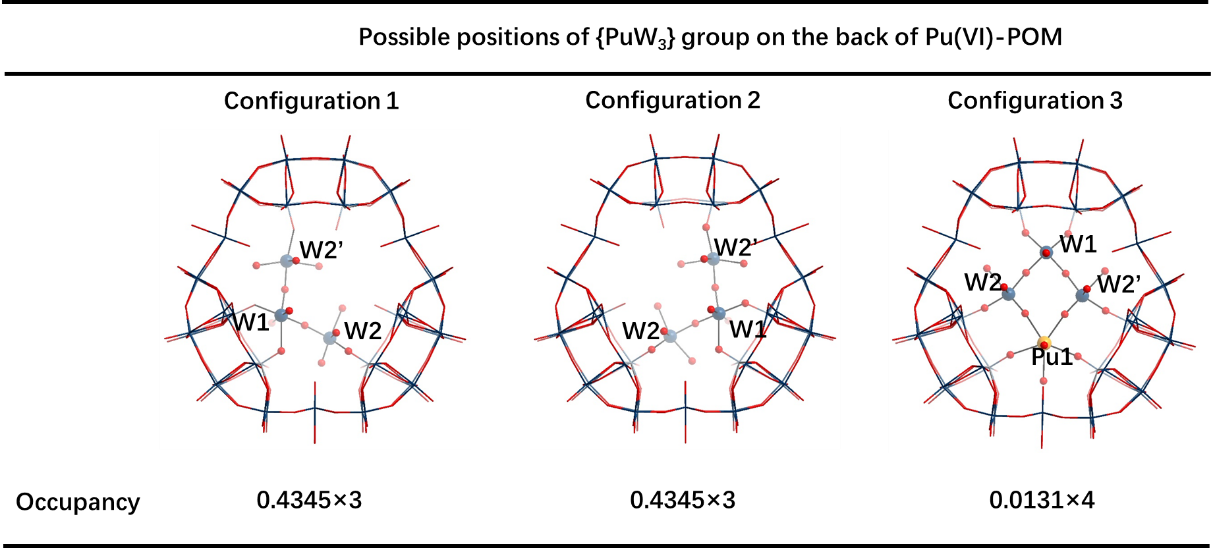


**Fig. 38** The positional disorder of {PuW_3_} group over three positions on the opposite face of Pu(VI)-POM.


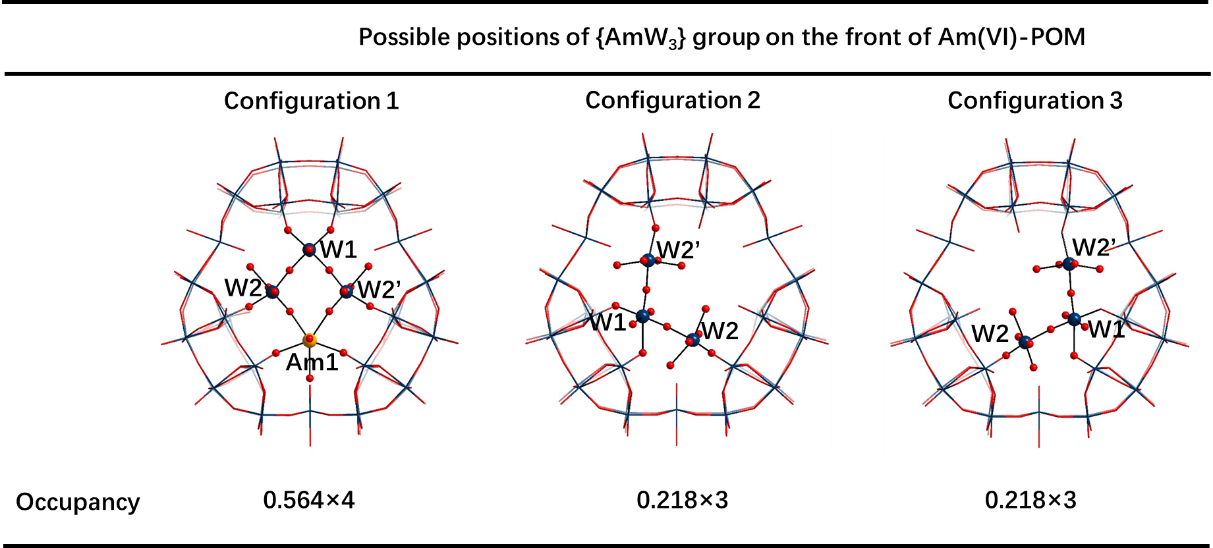


**Fig. 39** The positional disorder of {AmW_3_} group over three positions on the front of Am(VI)-POM.


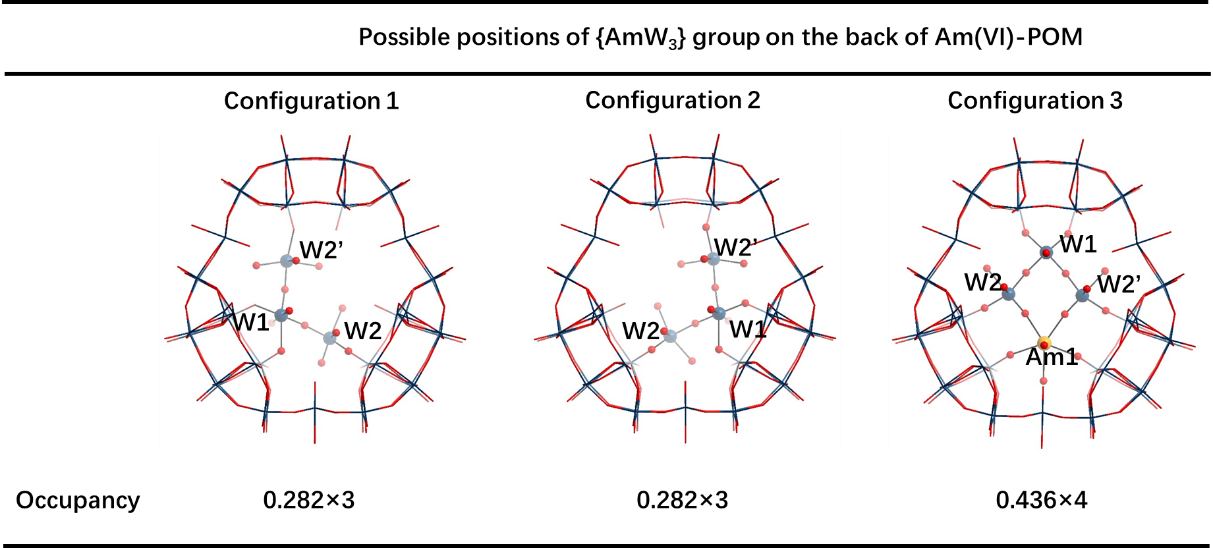


**Fig. 40** The positional disorder of {AmW_3_} group over three positions on the opposite face of Am(VI)-POM.

# Supplementary tables

**14. Table 1** Comparisons on the reported values on the binding constant (log*β*) between actinyl(VI) ions and inorganic ligands in aqueous solution.

| Reaction | log*β* | condition | reference |
| --- | --- | --- | --- |
| AmO_2_^2+^+{Se_6_W_45_} ⇌ {AmO_2_(Se_6_W_45_)} | 6.17 ± 0.10 | 0.1 M HNO_3_ | This work |
| PuO_2_^2+^+{Se_6_W_45_} ⇌ {PuO_2_(Se_6_W_45_)} | 7.03 ± 0.10 | 0.1 M HNO_3_ | This work |
| UO_2_^2+^+{SiW_12_} ⇌ {UO_2_(SiW_12_)} | 2.21 | 0.1 M NaClO_4_ | S12 |
| UO_2_^2+^+HP_2_W_17_^2-^ ⇌ UO_2_(HP_2_W_17_) | 3.9 | 0.1 M HClO_4_ | S11 |
| PuO_2_^2+^+HP_2_W_17_^2-^ ⇌ PuO_2_(HP_2_W_17_) | 3.3 | 0.1 M HClO_4_ | S11  S1 |
| NpO_2_^2+^+HP_2_W_17_^2-^ ⇌ PuO_2_(HP_2_W_17_) | 3.4 | 0.1 M HClO_4_ |  |
| NpO_2_^2+^+{SiW_11_} ⇌ {NpO_2_(SiW_11_)} | 3.8 | 0.1 M HClO_4_ |  |
| PuO_2_^2+^+{SiW_11_} ⇌ {PuO_2_(SiW_11_)}) | 3.5 | 0.1 M HClO_4_ |  |
| UO_2_^2+^+{PW_11_} ⇌ {UO_2_(PW_11_)} | 4.5 | 0.1 M HClO_4_ |  |
| NpO_2_^2+^+{PW_11_} ⇌ {NpO_2_(PW_11_)} | 4.2 | 0.1 M HClO_4_ |  |
| PuO_2_^2+^+{PW_11_} ⇌ {PuO_2_(PW_11_)} | 3.8 | 0.1 M HClO_4_ |  |
| UO_2_^2+^+F^-^ ⇌ UO_2_F^+^ | 4.6±0.02 | 1 mol/L NaClO_4_ |  |
| UO_2_^2+^+SO_4_^2-^ ⇌ UO_2_SO_4_ | 2.42 | 0.2 M NaClO_4_ | S2 |
| UO_2_^2+^+SO_4_^2-^ ⇌ UO_2_SO_4_  UO_2_^2+^+HSeO_3_^-^ ⇌ UO_2_(HSeO_3_)^+^ | 1.88 | 1 M NaClO_4_ | S2  S3 |
|  | 3.35±0.12 | 0.05 M NaClO_4_ |  |
| UO_2_^2+^+SeO_4_^2-^ ⇌ UO_2_SeO_4_ | 1.57±0.01 | 3 M NaClO_4_ | S4 |
| UO_2_^2+^+Si(OH)_4_ ⇌ UO_2_(OSi(OH)_3_)^+^ + H^+^ | -2.92±0.06 | 0.1 M NaClO_4_ | S5 |
| UO_2_^2+^+IO_3_^-^ ⇌ UO_2_(IO_3_)^+^ | 1.6±0.1 | 0.05 M NaClO_4_ | S6 |
| UO_2_^2+^+IO_4_^-^ ⇌ UO_2_(IO_4_)^+^ | 1.8±0.2 | 0.1 M NaClO_4_ | S7 |
| UO_2_^2+^+PO_4_^3-^ ⇌ UO_2_PO_4_^-^ | 11.894 | 0.5 M NaClO_4_ | S8 |
| UO_2_^2+^+HPO_4_^2-^ ⇌ UO_2_HPO_4_ | 6.353 | 0.5 M NaClO_4_ | S8  S9 |
| UO_2_^2+^+H_2_PO_4_^-^ ⇌ UO_2_H_2_PO_4_^+^ | 1.2 | 3 M HNO_3_ |  |
| UO_2_^2+^+NO_3_^-^ ⇌ UO_2_NO_3_^+^ | -0.62±0.04 | 1 M NaClO_4_ | S10 |

**15. Table 2** The change in luminescence lifetime (*τ*) and inner-sphere coordinated number (*N*_H2O_, with an uncertainty of ± 0.5) of Eu(III) during the titration.

| POM/Eu  ratio | 0 | 0.2 | 0.4 | 0.6 | 0.8 | 1.0 | 2.0 | 3.0 | 4.0 |
| --- | --- | --- | --- | --- | --- | --- | --- | --- | --- |
| *τ* (ms) | 0.117 | 0.126 | 0.134 | 0.141 | 0.146 | 0.149 | 0.162 | 0.169 | 0.173 |
| *N*_H2O_ | 8.56 | 7.88 | 7.36 | 7.00 | 6.75 | 6.57 | 6.03 | 5.77 | 5.62 |

**16. Table 3** Redox peak potentials for the tungsten or americium waves determined by cyclic voltammetry (V vs SCE).

|  | **E_p,Ox_ /V** | **E_p,Red_ /V** | **E_1/2_ /V** | **E_p/2,Ox_ /V** | **E_p/2,Red_ /V** |
| --- | --- | --- | --- | --- | --- |
| POM | -0.17  -0.25 | -0.13  -0.20 | -0.15  -0.22 |  |  |
| U(VI)-POM | -0.16  -0.24 | -0.11  -0.19 | -0.14  -0.22 |  |  |
| Am(VI) | 1.44 | 1.29 | 1.37 | 1.33 | 1.18 |
| Am(VI)-POM | 1.25 | 0.34 | 0.80 | 1.15 | 0.21 |

**17. Table 4** Selected bond lengths of An-O in An(VI)-POM.

| Bond length summary / Å | **U(VI)-POM** | **Np(VI)-POM** | **Pu(VI)-POM** | **Am(VI)-POM** |
| --- | --- | --- | --- | --- |
| An-O_yl_1 | 1.720(2) | 1.718(3) | 1.704(4) | 1.681(2) |
| An-O_yl_2 | 1.747(2) | 1.736(3) | 1.724(4) | 1.718(2) |
| An-O_eq_1 | 2.348(2) | 2.352(4) | 2.301(4) | 2.411(3) |
| An-O_eq_2 | 2.367(2) | 2.338(4) | 2.424(4) | 2.389(3) |
| An-O_eq_3 | 2.490(3) | 2.466(5) | 2.590(5) | 2.468(4) |

**18. Table 5** EDA-NOCV energies of An(VI)-POMs and Ln(H_2_O)_8_ at the PBE/TZ2P/TZP/DZP level.

|  | **U(VI)-POM** | **Np(VI)-POM** | **Pu(VI)-POM** | **Am(VI)-POM** | **Nd(H_2_O)_8_** | **Eu(H_2_O)_8_** |
| --- | --- | --- | --- | --- | --- | --- |
| E_int_ | -508.26 | -498.74 | -494.46 | -491.22 | -37.50 | -37.74 |
| E_pauli_ | 177.25 | 178.07 | 165.13 | 152.97 | 26.88 | 25.31 |
| E_elstat_ | -369.67 | -369.02 | -362.82 | -357.48 | -13.47 | -14.30 |
| E_orb_ | -315.84 | -307.79 | -296.77 | -286.71 | -24.03 | -23.44 |

# 19. BVS calculation results of POM and An-POM.

**Table 6** BVS calculation results of POM

| Oxygen Code | bond distance/Å | Bond Valence | Protonation Degree |
| --- | --- | --- | --- |
| O4S | 2.137 | 0.458 | 2 |
| O47 | 2.295 | 0.272 | 2 |
| O47’ | 2.295 | 0.272 | 2 |
| O4_1 | 2.271 | 0.294 | 2 |
| O6_1 | 2.298 | 0.269 | 2 |
| O6_1’ | 2.298 | 0.269 | 2 |
| O2_4 | 2.286 | 0.281 | 2 |
| O11_4 | 2.154 | 0.433 | 2 |
| O7_4 | 2.154 | 0.433 | 2 |
| Total 9 H_2_O | | | |

**Table 7** BVS calculation results of U(VI)-POM

| Oxygen Code | bond distance/Å | Bond Valence | Protonation Degree |
| --- | --- | --- | --- |
| O1 | 2.325 | 0.246 | 2 |
| O51 | 2.327 | 0.245 | 2 |
| O51’ | 2.327 | 0.245 | 2 |
| O1_1 | 2.280 | 0.286 | 2 |
| O81 | 2.223 | 0.345 | 2 |
| O81’ | 2.223 | 0.345 | 2 |
| O5_4 | 2.300 | 0.267 | 2 |
| O1_4 | 2.108 | 0.505 | 2 |
| O9_4 | 2.261 | 0.304 | 2 |
| O8_1 | 2.490 | 0.340 | 2 |
| Total 10 H_2_O | | | |

**Table 8** BVS calculation results of Np(VI)-POM

| Oxygen Code | bond distance/Å | Bond Valence | Protonation Degree |
| --- | --- | --- | --- |
| O1 | 2.232 | 0.335 | 2 |
| O51 | 2.238 | 0.328 | 2 |
| O51’ | 2.238 | 0.328 | 2 |
| O1_1 | 2.147 | 0.444 | 2 |
| O81 | 2.171 | 0.410 | 2 |
| O81’ | 2.171 | 0.410 | 2 |
| O5_4 | 2.292 | 0.275 | 2 |
| O1_4 | 2.224 | 0.344 | 2 |
| O9_4 | 2.127 | 0.474 | 2 |
| O8_1 | 2.466 | 0.360 | 2 |
| Total 10 H_2_O | | | |

**Table 9** BVS calculation results of Pu(VI)-POM

| Oxygen Code | bond distance/Å | Bond Valence | Protonation Degree |
| --- | --- | --- | --- |
| O1 | 2.214 | 0.355 | 2 |
| O51 | 2.225 | 0.343 | 2 |
| O51’ | 2.225 | 0.343 | 2 |
| O1_1 | 2.196 | 0.377 | 2 |
| O81 | 2.161 | 0.423 | 2 |
| O81’ | 2.161 | 0.423 | 2 |
| O5_4 | 2.302 | 0.266 | 2 |
| O1_4 | 2.216 | 0.353 | 2 |
| O11_4 | 2.361 | 0.219 | 2 |
| O8_1 | 2.59 | 0.268 | 2 |
| Total 10 H_2_O | | | |

**Table 10** BVS calculation results of Am(VI)-POM

| Oxygen Code | bond distance/Å | Bond Valence | Protonation Degree |
| --- | --- | --- | --- |
| O1 | 2.284 | 0.282 | 2 |
| O51 | 2.309 | 0.260 | 2 |
| O51’ | 2.309 | 0.260 | 2 |
| O1_1 | 2.211 | 0.359 | 2 |
| O81 | 2.299 | 0.268 | 2 |
| O81’ | 2.299 | 0.268 | 2 |
| O5_4 | 2.145 | 0.446 | 2 |
| O1_4 | 2.265 | 0.300 | 2 |
| O10_4 | 2.230 | 0.337 | 2 |
| O8_1 | 2.468 | 0.358 | 2 |
| Total 10 H_2_O | | | |

**20. Table 11** The results of CHN elemental analysis for POM and U(VI)-POM.

| Element Weight % | C | N | H |
| --- | --- | --- | --- |
| POM | 3.18 | 1.68 | 1.34 |
| U(VI)-POM | 3.37 | 1.60 | 1.41 |
| U(VI)-POM-2 | 0.76 | 0.72 | 0.99 |

**21. Table 12** Crystallographic data and structure refinement parameters for POM and An(VI)-POM.

| Compound | POM | U(VI)-POM | Np(VI)-POM | Pu(VI)-POM | Am(VI)-POM |
| --- | --- | --- | --- | --- | --- |
| T(K) | 120.0 | 296.15 | 120.0 | 120.0 | 120.0 |
| Space group | *P*2_1_/*m* | *P*2_1_/*m* | *P*2_1_/*m* | *P*2_1_/*m* | *P*2_1_/*m* |
| a(Å) | 15.6207(6) | 13.5255(6) | 13.9550(12) | 13.6840(11) | 13.5104(13) |
| b(Å) | 33.5801(11) | 33.5417(12) | 33.429(3) | 33.370(2) | 33.262(3) |
| c(Å) | 20.8294(7) | 22.7478(8) | 22.8155(18) | 22.6804(16) | 22.8592(19) |
| α(deg) | 90 | 90 | 90 | 90 | 90 |
| β(deg) | 98.130(2) | 95.5950(10) | 96.025(4) | 96.276(3) | 95.555(4) |
| γ(deg) | 90 | 90 | 90 | 90 | 90 |
| V(Å^3^) | 10816.1(7) | 10270.8(7) | 10584.6(15) | 10294.7(13) | 10224.4(16) |
| Z | 2 | 2 | 2 | 2 | 2 |
| F(000) | 9745 | 10004 | 10006 | 10008 | 10031 |
| Independent reflections | 19425 | 18444 | 18996 | 17350 | 18287 |
| R_int_ | 0.0725 | 0.0974 | 0.1558 | 0.1800 | 0.0716 |
| Completeness | 99.8% | 99.9% | 99.6% | 99.1% | 99.4% |
| Goodness-of-fit | 1.038 | 1.080 | 1.035 | 1.034 | 1.040 |
| R_1_[I > 2σ(I)] | 0.0680 | 0.0943 | 0.1306 | 0.1208 | 0.0886 |
| wR_2_[I > 2σ(I)] | 0.1793 | 0.1861 | 0.2915 | 0.2786 | 0.2430 |
| R_1_[all data] | 0.0909 | 0.1361 | 0.1836 | 0.2095 | 0.0946 |
| wR_2_[all data] | 0.2002 | 0.2049 | 0.3220 | 0.3332 | 0.2476 |

R = Σ||F_o_|-|F_c_|| / Σ|F_o_|; wR = {Σ[w(|F_o_|^2^ - |F_c_|^2^)^2^] / Σ[w(|F_o_|^4^)]}^1/2^

# 22. References

S1. Tian, G. & Rao, L. Effect of temperature on the complexation of Uranium (VI) with fluoride in aqueous solutions. *Inorg. Chem.* **48**, 6748-6754 (2009).

S2. Geipel, G. *et al.* Uranium (VI) sulfate complexation studied by time-resolved laser-induced fluorescence spectroscopy (TRLFS). *Radiochim. Acta* **75**, 199-204 (1996).

S3. Sladkov, V., Fourest, B. & Mercier, F. Uranyl-Se (IV) interaction in aqueous acid solutions studied by time-resolved laser-induced fluorescence spectroscopy (TRLFS) and UV-Vis spectrophotometry. *Dalton Trans.* **37***,* 7734 (2009).

S4. Lubal, P. & Havel, J. The study of complex equilibria of uranium (VI) with selenate. *Talanta* **44**, 457-466 (1997).

S5. Jensen, M. P. & Choppin, G. R. Complexation of uranyl (VI) by aqueous orthosilicic acid. *Radiochim. Acta* **82**, 83-88 (1998).

S6. Karbowiak, M., Fourest, B., Hubert, S. & Moulin, C. Complexation of U (VI) with iodate ions: determination of stability constants by using spectroscopic methods and capillary zone electrophoresis. *Radiochim. Acta* **91**, 505-512 (2003).

S7. Karbowiak, M., Hubert, S., Fourest, B. & Moulin, C. Complex formation of uranium (VI) in periodate solutions. *Radiochim. Acta* **92**, 489-494 (2004).

S8. Romero-González, M. R., Cheng, T., Barnett, M. O. & Roden, E. E. Surface complexation modeling of the effects of phosphate on uranium (VI) adsorption. *Radiochim. Acta* **95**, 251-259 (2007).

S9. Koma, Y., Aoshima, A., Kamoshida, M. & Sasahira, A. Extraction of Am (VI) from nitric acid solution containing phosphate anion by TBP. *J. Nucl. Sci. Technol.* 39, 317-320 (2002).

S10. Rao, L. & Tian, G. Thermodynamic study of the complexation of uranium (VI) with nitrate at variable temperatures. *J. Chem. Thermodynamics* **40**, 1001-1006 (2008).

S11. Yusov, A. B. & Shilov, V. P. Interaction of U (VI), Np (VI), and Pu (VI) ions with unsaturated heteropolytungstates of the 17th and 11th series in aqueous solutions. *Radiochem.* **49**, 144-151 (2007).

S12. Saito, A. & Choppin, G. R. Interaction of Metal Cations with Heteropolytungstate Ions SiW_12_O_40_^4-^ and P_2_W_18_O_62_^6-^. *Inorg. Chem*. **30**, 4563-4566 (1991).
